# Hydrophilic Ethylene Glycol Fragments: A Determinant Affecting the Therapeutic Index of Paclitaxel Prodrug Nanoassemblies

**DOI:** 10.1021/acscentsci.4c01004

**Published:** 2024-11-20

**Authors:** Yaqi Li, Yixin Sun, Qing Wang, Shuo Wang, Cuiyun Liu, Yuetong Huang, Wenxin Zhong, Xiyan Wang, Wenjing Wang, Shiyi Zuo, Xianbao Shi, Xiaohui Pu, Jin Sun, Zhonggui He, Bingjun Sun

**Affiliations:** † Department of Pharmaceutics, Wuya College of Innovation, Shenyang Pharmaceutical University, Shenyang, 110016, China; ‡ Joint International Research Laboratory of Intelligent Drug Delivery Systems, Ministry of Education, Shenyang 110016, China; § State Key Laboratory of Antiviral Drugs, School of Pharmacy, Henan University, N. Jinming Avenue, Kaifeng 475004, China; ∥ Department of Pharmacy, The First Affiliated Hospital of Jinzhou Medical University, Jinzhou 121001, China; ⊥ School of Chemical Engineering, The University of Adelaide, Adelaide, South Australia 5005, Australia

## Abstract

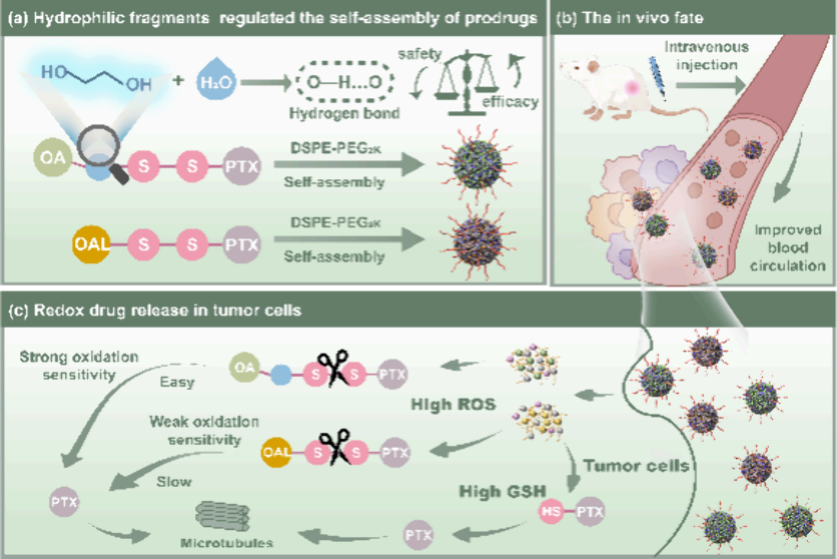

Prodrug-based nanoassemblies are promising platforms for cancer therapy. Prodrugs typically consist of three main components: drug modules, intelligent response modules, and modification modules. However, the available modification modules are usually hydrophobic aliphatic side chains, which affect the activation efficiency of the prodrugs. Herein, hydrophilic ethylene glycol fragments were inserted between the modification modules and the response modules, and the important effects of hydrophilic fragments on the assembly, drug release, and therapeutic index of the prodrugs were investigated. Notably, the introduction of hydrophilic fragments affected the intermolecular forces of the prodrugs and increased the interaction of hydrogen bonding. In addition, hydrophilic fragments significantly improved the redox drug release profiles, which affected the therapeutic index of the prodrug nanoassemblies. PTX-SS-OA NPs with hydrophilic fragments exhibited increased redox sensitivity, enhanced cytotoxicity, and superior antitumor efficacy. In comparison, PTX-SS-OAL NPs without hydrophilic fragments showed limited redox sensitivity and cytotoxicity but displayed better safety. Overall, the hydrophilic fragment is a critical determinant in modulating the therapeutic index of the prodrug nanoassemblies, which contributes to the development of advanced prodrug nanodelivery systems.

## Abstract 2

Hydrophilic ethylene glycol fragment is a critical determinant in modulating the therapeutic index of the paclitaxel prodrug nanoassemblies.

## Introduction

Chemotherapy contributes to be one of the predominant therapeutic strategies employed in clinical cancer treatment. However, its clinical application is limited by the adverse physicochemical properties and low delivery efficiency of the therapeutic agents.^1−3^ For instance, paclitaxel (PTX), a first-line broad-spectrum chemotherapeutic agent, has shown potent antitumor activity.^4,5^ Nevertheless, the poor water solubility of paclitaxel (less than 0.4 μg/mL) poses a challenge in its formulation. The commercially available injection Taxol addresses this issue by utilizing Cremophor EL and ethanol as solubilizers, resulting in low bioavailability and significant side effects, which restrict its clinical application.^6−8^ To improve the therapeutic index of PTX, various nanoparticle-based delivery systems have been extensively studied.^9,10^ Among them, Abraxane is the unique FDA-approved PTX nanoparticle formed by encapsulating PTX within the hydrophobic core of human serum albumin (HSA).^11,12^ The hydrophobic interaction of PTX with HSA significantly improves the solubility and tolerability of PTX.^11,12^ However, the complexity of the preparation process, the easy contamination of HSA, and the poor tumor selectivity restrict the clinical application of Abraxane.^9,13^

Recently, small-molecule prodrug nanoassemblies have been proposed to reduce the complexity of nanocarriers.^14−16^ Small-molecule prodrug nanoassemblies, which utilize prodrug strategies improving the physicochemical properties of the parent drug and forming nanoparticles by self-assembly of the prodrugs, are simple to prepare and possess ultrahigh drug loading.^17,18^ Prodrugs usually comprise drug modules, intelligent response modules, and modification modules. As a stimulus-responsive drug delivery system, prodrug nanoassemblies with intelligent response modules could be selectively triggered at the tumor sites to release the parent drug.^19−21^ Abnormal tumor growth leads to the development of a tumor-specific microenvironment characterized by elevated redox levels, hypoxia, slightly acidic pH, which lay the cornerstone for the design of tumor-selective drug delivery systems.^22^ Since the concentration of reactive oxygen species (ROS) and glutathione (GSH) in tumor cells is 100 and 7–10 times higher than that in normal cells, the design of redox-responsive modules would achieve tumor-selective activation of prodrugs.^23−27^ Disulfide bonds with dual redox sensitivity are promising intelligent response modules for clinical development.^28−31^

In terms of modification modules, hydrophobic aliphatic side chains have been extensively studied.^32−36^ However, short carbon chain lengths reduce the stability of the prodrug nanoassemblies and exhibit undesirable pharmacokinetics, while long carbon chain lengths compromise the activation of the prodrugs.^37,38^ It was shown that hydrophilic fragments can act as hydrogen bond donors or acceptors, forming hydrogen bonds with water molecules and influencing the hydrolysis of adjacent chemical bonds.^39^ Ethylene glycol, a hydrophilic compound containing two hydroxyl groups, is widely used as a linker in the structural design of prodrugs.^40^ Therefore, we speculated whether the insertion of hydrophilic fragments between the modification modules and the response modules could solve the above problem.

**Scheme 1:**
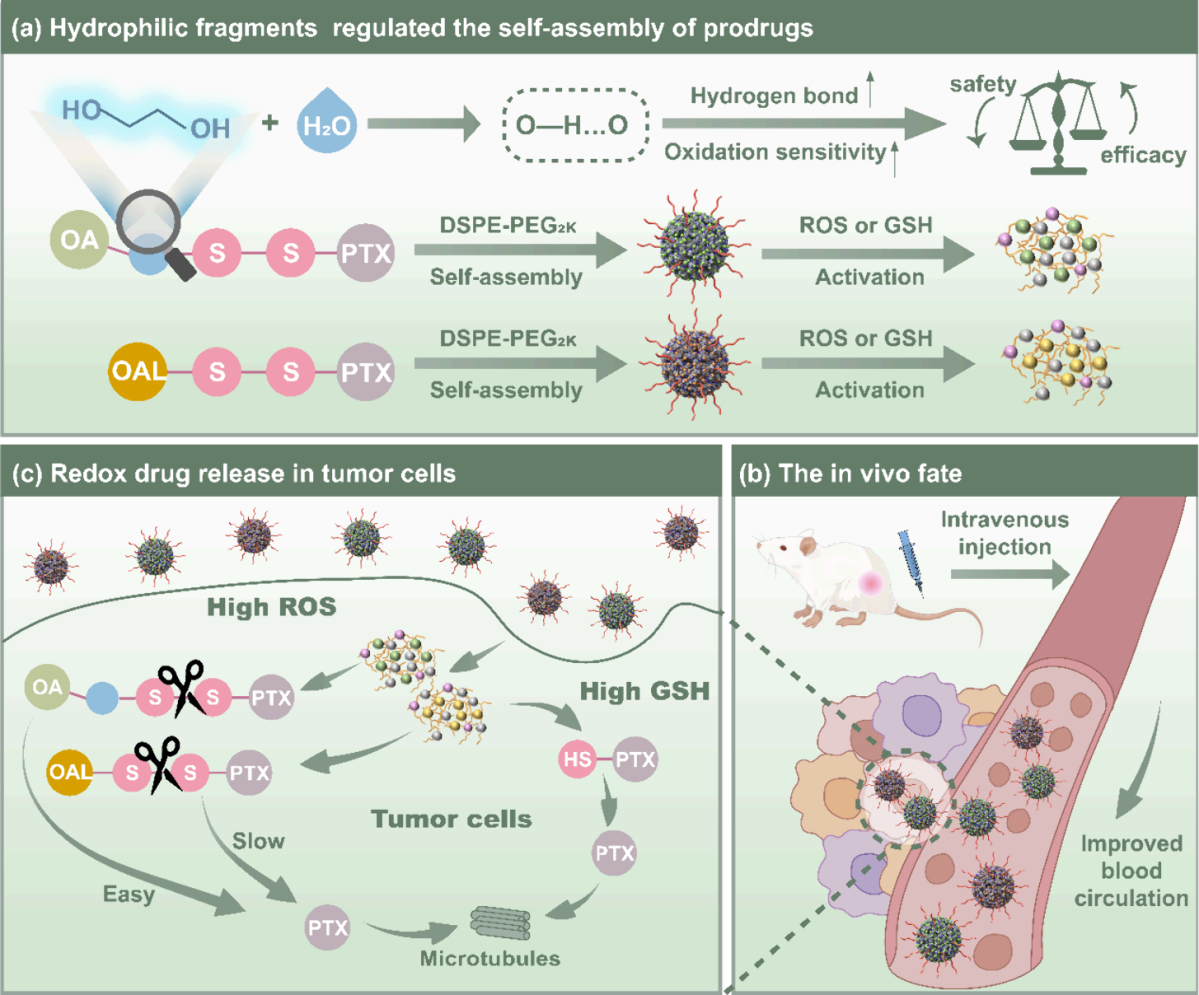
Schematic Illustrations: Hydrophilic Fragment Is a Critical Determinant in Modulating the Therapeutic Index of the Paclitaxel Prodrug Nanoassemblies

To validate our hypothesis, two prodrugs (PTX-SS-OA and PTX-SS-OAL) were constructed using hydrophilic ethylene glycol as the variable, PTX as the drug module, a disulfide bond as the redox-responsive module, and oleic acid (OA) or oleyl alcohol (OAL) as the modification module. The presence of hydrophilic fragments significantly influenced the assembly performance, drug release behavior, and therapeutic index of prodrug nanoassemblies (Scheme 1): (i) PTX-SS-OA, containing ethylene glycol, exhibited stronger intermolecular hydrogen bonding forces, driving the stable assembly. (ii) The introduction of ethylene glycol accelerated the release of PTX from the prodrug nanoassemblies. As a result, PTX-SS-OA NPs with ethylene glycol exhibited higher redox sensitivity, enhanced cytotoxicity, and antitumor efficacy. (iii) PTX-SS-OAL NPs, lacking ethylene glycol, showed limited redox sensitivity and cytotoxicity but displayed better safety. Thus, the hydrophilic fragment is a critical determinant in modulating the therapeutic index of the prodrug nanoassemblies.

## Results and Discussion

### Synthesis of Prodrugs

The synthesis of the two prodrugs is shown in Figure S1. The ethylene glycol-containing prodrug was PTX-SS-OA, and the ethylene glycol-free prodrug was PTX-SS-OAL. The confirmation of the chemical structures of the prodrugs was achieved through ^1^H NMR (Figure S2 and Figure S3). PTX-SS-OA and PTX-SS-OAL were subjected to HRMS to ensure their molecular weight (Figure S2 and Figure S3). Moreover, the prodrugs exhibited over 99% purity, as determined by HPLC (Figure S2 and Figure S3), which satisfied the requirements of the following experiments.

### Fabrication and Characterization of PTX Prodrugs Nanoassemblies (PPNAs)

**Figure 1:**
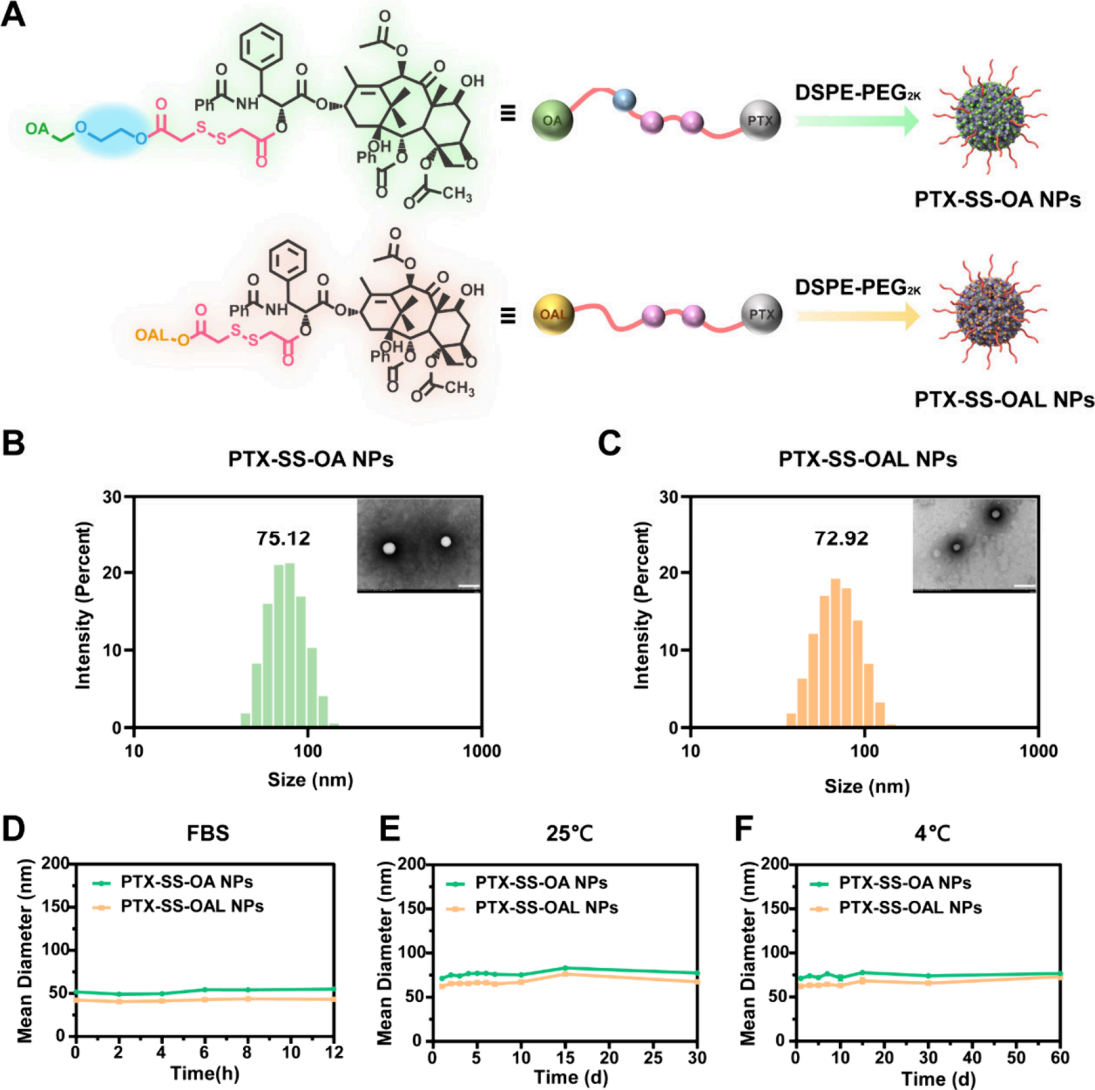
Characterization and stability of PPNAs. (A) Prodrug chemical structures. (B–C) Particle size distribution and TEM images of PEGylated PPNAs. Scale bar: 200 nm. (D) The colloidal stability of PEGylated PPNAs. (E–F) Storage stability of PEGylated PPNAs.

For the preparation of PEGylated PPNAs, DSPE-PEG_2K_ was used as the surface modifier to improve the stability and prolong the blood circulation time of the PPNAs. The PPNAs were named PTX-SS-OA NPs and PTX-SS-OAL NPs (Figure 1A and Table S1). Both PEGylated PPNAs had a diameter of approximately 70 nm. The negative surface charge (zeta potential was about −20 mV) of PPNAs effectively prevented the aggregation of nanoassemblies by inducing charge repulsion, thus improving their colloidal stability. As shown in Table S1, the drug loadings of the PPNAs were 51.54% (PTX-SS-OA NPs) and 53.89% (PTX-SS-OAL NPs). The spherical structures of the PPNAs were observed by TEM (Figure 1B–C).

### Colloidal Stability

The PEGylated PPNAs were coincubated with PBS containing 10% FBS to explore the colloidal stability. As shown in Figure 1D, the PPNAs revealed excellent colloidal stability evidenced by the negligible change in particle sizes observed over 12 h. Moreover, to study the stability of storage, PPNAs were stored at 25 °C (room temperature) and 4 °C. The PPNAs remained stable for 30 days (25 °C) and 60 days (4 °C), respectively (Figure 1E–F). As shown in Figure S14, the PEGylated PPNAs exhibited preeminent stability in 0.9% NaCl after incubation for 24 h, indicating that PEGylated PPNAs could be used as an intravenous preparation. These findings indicated that DSPE-PEG_2K_ modification endowed nanoassemblies with good stability, which was beneficial in improving the pharmacokinetics of PTX.

### Self-Assembly Mechanism

The non-PEGylated PPNAs with a negative surface charge of −16 mV were selected to compare the self-assembly capacity and mechanism of the prodrugs (Table S3). The prodrugs could not assemble into nanoassemblies and precipitated immediately when dropped into deionized water at a concentration of 0.5 mg/mL (Figure S4A). When the concentration was reduced to 0.2 and 0.1 mg/mL, both prodrugs could assemble to form nanoassemblies, while PTX-SS-OA NPs showed smaller particle sizes (Figure S4A, Figure S5 and Tables S2–S3). This result indicated that the introduction of hydrophilic ethylene glycol improved the self-assembly capacity of prodrugs when the structure of the modification modules was almost the same.

Molecular simulations were performed to confirm the self-assembly mechanism of the prodrugs. As illustrated in Figure S4C–D, π–π stacking, hydrogen bonding forces, and hydrophobic interaction were involved in the self-assembly process of prodrugs. The presence of hydrogen bonds in the two prodrugs was confirmed by the results of infrared spectroscopy, as the wide peak deformation of the O–H stretching vibration at a wavelength of about 3510 nm in the two prodrugs (Figure S4E–G). To further investigate whether there were differences in interactions between PTX-SS-OA and PTX-SS-OAL, the non-PEGylated PPNAs were diluted with urea (a hydrogen bond blocker), sodium dodecyl sulfate (SDS, a disruptor of hydrophobic interaction), and sodium chloride (an ionic competitive agent) respectively. The results are depicted in Figure S4H–J. After incubation in urea solution for 24 h, the particle sizes of PTX-SS-OA NPs increased three times, but the size change of PTX-SS-OAL NPs was negligible, indicating that the hydrogen bonding forces in PTX-SS-OA NPs were stronger than PTX-SS-OAL NPs (Figure S4H). This phenomenon could be attributed to the existence of ethylene glycol in PTX-SS-OA NPs, which increased the number of hydrogen bond receptors in the prodrug molecules, thereby promoting the formation of hydrogen bonds between the PTX-SS-OA molecules. After incubation with SDS for 1 h, both PTX-SS-OAL NPs and PTX-SS-OA NPs experienced dramatic changes in particle sizes, especially for PTX-SS-OAL NPs (Figure S4I and Figure S6). The instability of nanoassemblies in SDS were related to the strength of hydrophobic interactions. The hydrophobicity of the prodrugs was assessed by calculating their oil–water partition coefficient (Log*P*). As displayed in Figure S4B, the Log*P* values of PTX-SS-OA and PTX-SS-OAL were 11.259 and 11.686, respectively. Therefore, the hydrophobicity and hydrophobic interactions of PTX-SS-OA were weaker than those of PTX-SS-OAL (Figure S4B, Figure S2C, and Figure S3C). Furthermore, the size of PTX-SS-OA NPs increased significantly after incubation in sodium chloride for 2 h, and the size of PTX-SS-OAL NPs enlarged significantly after incubation for 8 h, confirming the existence of an electrostatic interaction in non-PEGylated PPNAs (Figure S4J).

In summary, π–π stacking, hydrogen bonding forces, hydrophobic interaction, and electrostatic interaction participated in the self-assembly of prodrugs. Among them, PTX-SS-OA NPs exhibited stronger intermolecular hydrogen bonding forces due to the presence of ethylene glycol, while PTX-SS-OAL NPs exhibited stronger intermolecular hydrophobic interactions. In comparison to non-PEGylated PPNAs, PEGylated PPNAs exhibited enhanced stability. As a result, subsequent experiments were conducted using the PEGylated PPNAs.

### Redox Dual-Sensitive Drug Release

**Figure 2:**
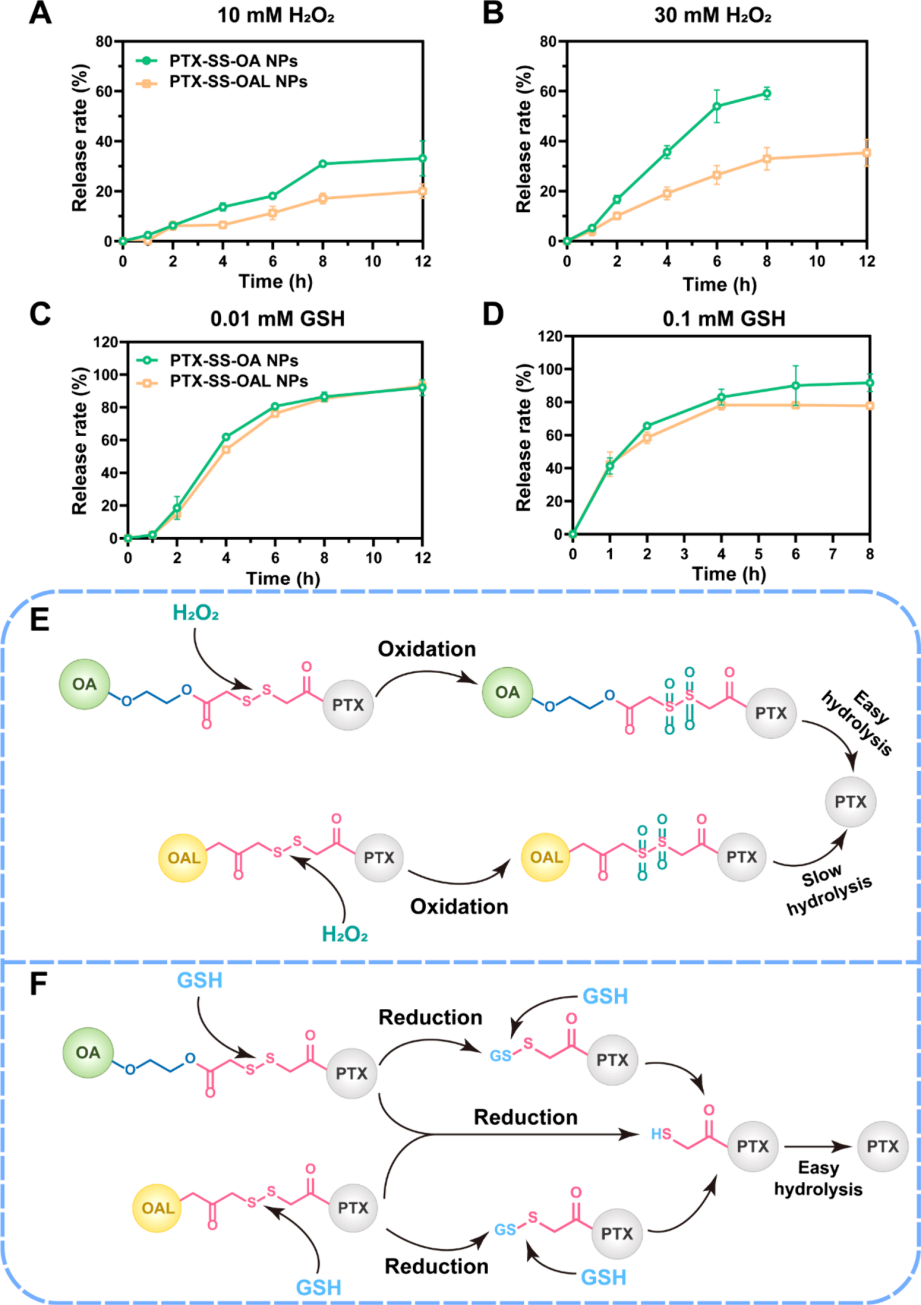
Redox dual-sensitive drug release. PTX release profiles with (A) 10 mM H_2_O_2_, (B) 30 mM H_2_O_2_, (C) 0.01 mM GSH, and (D) 0.1 mM GSH. (E) Oxidization release mechanism of PPNAs. (F) Reduction release mechanism of PPNAs.

The redox dual-sensitive drug release was explored by using PBS containing H_2_O_2_ or GSH as the release medium. UPLC-MS-MS was used to detect the redox release intermediates (Figure S7A–D). Less than 10% of PTX was released by PPNAs within 12 h in release media without H_2_O_2_ or GSH (Figure S8). The designed prodrugs would undergo a stimulus response process when exposed to H_2_O_2_ or GSH, which could realize the tumor-targeted drug release.^19^ In the presence of H_2_O_2_, the disulfide bond was oxidized to sulfones or sulfoxides with enhanced hydrophilicity. This promoted the hydrolysis of adjacent ester bonds to release PTX, while hydrophilic ethylene glycol in the prodrug expedited the hydrolysis (Figure 2E). As illustrated in Figure 2A–B, the release rate of PTX from PPNAs in the medium containing different concentrations of H_2_O_2_ was PTX-SS-OA NPs > PTX-SS-OAL NPs. The main reason for the disparity was the hydrophilicity of oxidation intermediates. In PTX-SS-OA NPs, the oxidation intermediates were more hydrophilic due to the presence of ethylene glycol (Figure S7E–F). Consequently, PTX-SS-OA NPs were more easily hydrolyzed, which displayed stronger oxidative sensitivity than PTX-SS-OAL NPs.

The disulfide bond was reduced to thiol intermediates in the presence of GSH (Figure 2F). As in the oxidation process, hydrophilic thiol intermediates also promoted the hydrolysis of ester bonds and the release of PTX (Figure S7G). Since intermediates produced under reducing conditions were the same for both PPNAs, there was no difference in reduction release between the two PPNAs (Figure 2C–D).

### Cellular Uptake

**Figure 3:**
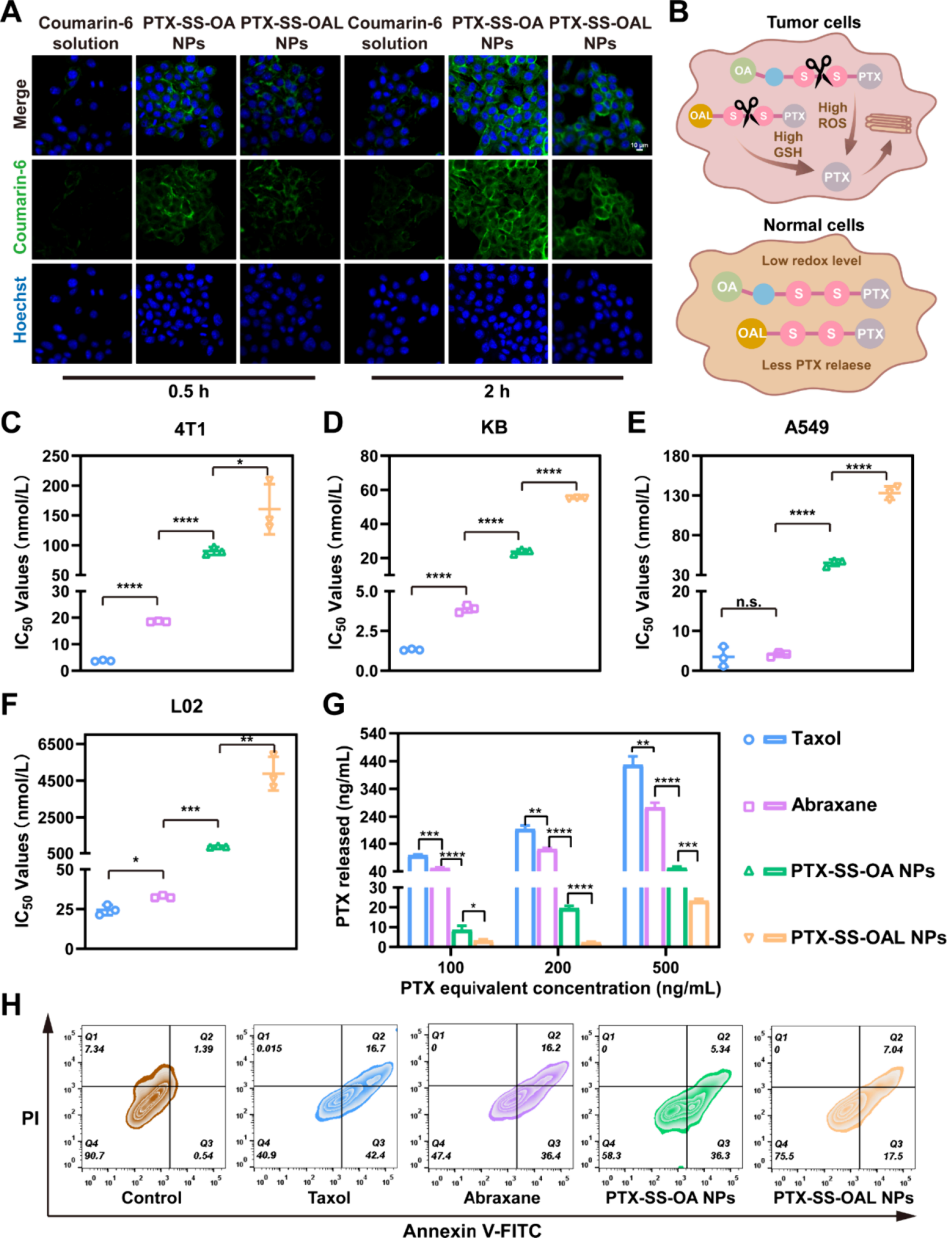
Cell assays of the PEGylated PPNAs. (A) Cellular uptake of coumarin-6 solution and coumarin-6 labeled PPNAs. Scale bar: 10 μm. (B) Tumor-selective bioactivation of PPNAs. The IC_50_ of Taxol, Abraxane, and PPNAs in (C) 4T1 cells, (D) KB cells, (E) A549 cells, and (F) L02 cells. (G) PTX released by different concentrations of PPNAs. (H) Cell apoptosis assay of Taxol, Abraxane, and PPNAs in 4T1 cells.

The successful uptake of PPNAs by tumor cells was significant in determining the effectiveness of prodrugs in combating tumor cells. In CLSM images, the intensity of green fluorescence was positively correlated with the uptake efficiency (Figure 3A). The cells treated with coumarin-6 labeled PPNAs exhibited a higher intensity of green fluorescence compared with coumarin-6 solution (Figure 3A). Moreover, the uptake of PPNAs by cells was contingent upon time, indicating a time-dependent process. The CLSM images were quantitatively analyzed using ImageJ software (Figure S9). Interestingly, after cultivation for 0.5 h, the green fluorescence intensity of two PPNAs was comparable, while after cultivation for 2 h, the cells treated with coumarin-6 labeled PTX-SS-OA NPs exhibited stronger fluorescence intensity. This phenomenon could be explained by the aggregation-caused quenching (ACQ) effect. The fluorescence of coumarin-6 was quenched due to the aggregation of the nanoassemblies. PTX-SS-OA NPs were more sensitive to oxidative conditions and could promote the disassembly of the nanoassemblies, thereby restoring the fluorescence intensity of coumarin-6.

### Cytotoxicity and Cell Apoptosis

The cytotoxicity of PPNAs against three tumor cell lines (4T1 cells, KB cells, and A549 cells) and one normal cell line (L02 cells) was investigated in this section. The order of the cytotoxicity of formulations was Taxol > Abraxane > PTX-SS-OA NPs > PTX-SS-OAL NPs in tumor cell lines (Figure 3C–E and Table S4). The reduced cytotoxicity of the PPNAs was attributed to the delayed release of PTX compared to that of Taxol and Abraxane. For PPNAs, the lower IC_50_ values of PTX-SS-OA NPs demonstrated higher cytotoxicity due to the presence of ethylene glycol which increased the oxidative sensitivity of PTX-SS-OA NPs and promoted more intracellular release of PTX. The intracellular drug release assay confirmed this conclusion (Figure 3G).

In normal cells, the cytotoxicity of PPNAs significantly declined (Figure 3F). The tumor selectivity index (SI) was calculated, and the results showed that PPNAs exhibited more significant tumor selectivity compared to Taxol and Abraxane (Table S5). It was demonstrated that the PPNAs could be activated specifically in the microenvironment of tumor tissue to release PTX, while remaining in a safer prodrug form in normal cells (Figure 3B).

In addition, cell apoptosis induced by Taxol, Abraxane and PPNAs was studied by a flow cytometer. As displayed in Figure 3H, the Q1 and Q4 regions represented the proportion of necrotic cells and surviving cells respectively. The apoptosis rate of cells was the sum of the values in the Q2 region (the proportion of late apoptotic cells) and the Q3 region (the proportion of early apoptotic cells). Apoptosis rates of Taxol and Abraxane treated 4T1 cells were 59.1% and 52.6%, respectively. The apoptosis rates induced by PPNAs were 41.64% (PTX-SS-OA NPs) and 24.54% (PTX-SS-OAL NPs), respectively, which were lower than those of Taxol and Abraxane. The results were in agreement with those of the cytotoxicity assays.

### Microtubule Depolymerization Inhibition

**Figure 4:**
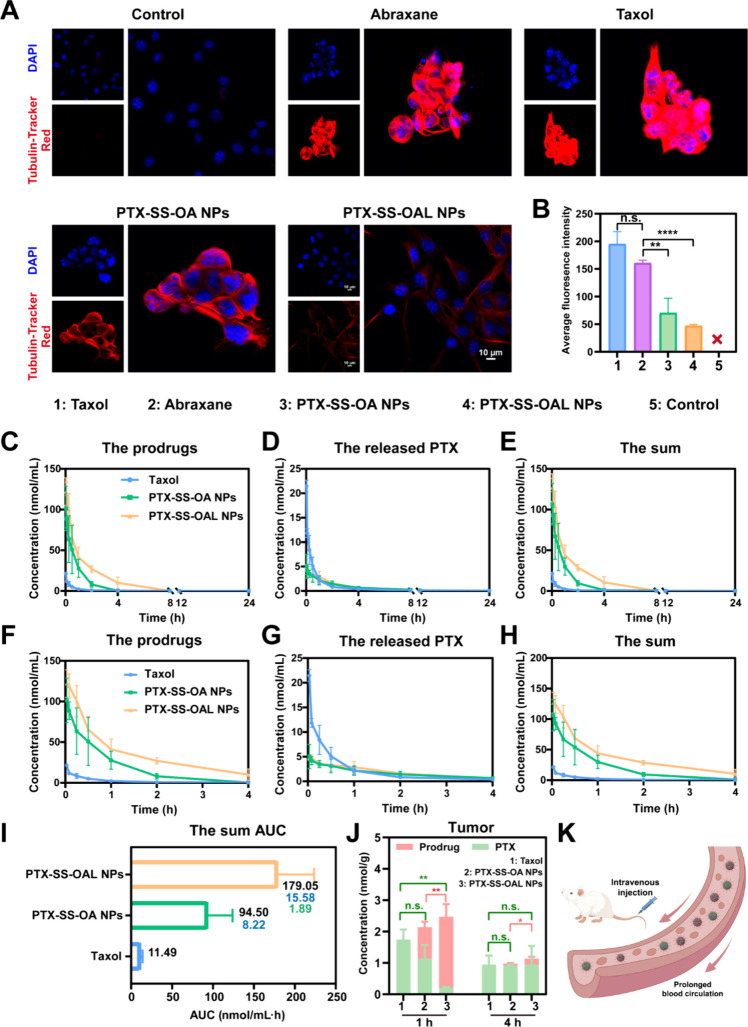
Microtubule depolymerization inhibition and pharmacokinetics of PEGylated PPNAs. (A) Microtubule depolymerization inhibitory effect of Taxol, Abraxane, and PPNAs. Scale bar: 10 μm. (B) Red fluorescence quantitative results of microtubule depolymerization inhibition assay. “**×**” represents that the red fluorescence intensity is too weak to be calculated. (C–E) Drug concentration–time curves (0–24 h) and (F–H) drug concentration–time curves (0–4 h) of the prodrugs, the released PTX, and the sum (prodrug and PTX, PTX equivalent). (I) AUC of the sum (prodrug and PTX, PTX equivalent). (J) Tumor accumulation of PPNAs. The green symbol represents the difference between the PTX released by each group, and the red symbol represents the difference of prodrug concentrations. (K) PPNAs in blood circulation.

PTX could promote tubulin polymerization by binding to tubulin, thus destroying cell mitosis and leading to cell apoptosis.^41^ The microtubule depolymerization inhibition caused by Taxol, Abraxane, and PEGylated PPNAs was explored in this section. Tubulin-Tracker Red was used to label the microtubules of the 4T1 cells. The nuclei were stained by DAPI. The red fluorescence intensity was significantly stronger in Taxol and Abraxane treated cells than in PPNAs treated cells (Figure 4A). The red fluorescence intensity was quantitatively analyzed with ImageJ software (Figure 4B). Obviously, the inhibitory capacity of Taxol and Abraxane on microtubule depolymerization was the strongest. The inhibition of microtubule depolymerization by PPNAs was weaker than that of Taxol and Abraxane. Among them, the inhibition effect of PTX-SS-OA NPs was slightly stronger than PTX-SS-OAL NPs, which was probably attributed to more PTX released by PTX-SS-OA NPs.

### Pharmacokinetics and Plasma Stability

Before the *in vivo* experiments, a hemolysis assay of PPNAs was conducted. As shown in Figure S10 and Table S6, PPNAs did not induce hemolysis, and the hemolysis percentage was extremely small, much less than 5%, meeting the requirement for intravenous injection. To further investigate the effect of ethylene glycol on the *in vivo* fate of PPNAs, a pharmacokinetic study of PPNAs and Taxol was conducted. Plasma samples were processed using the protein precipitation method (Figure S11F). The drug concentration–time curves (0–24 h and 0–4 h) were displayed in Figure 4C–H. The relevant pharmacokinetic parameters could be found in Table S7. As illustrated in Figure 4C–H, Taxol was observed to be rapidly cleared from the plasma, while PPNAs significantly prolonged the circulation time of PTX (Figure 4K). The sum of the areas under the concentration–time curves (AUC_0–24 h_) increased 8.22-fold and 15.58-fold for PTX-SS-OA NPs and PTX-SS-OAL NPs compared with Taxol, respectively (Figure 4I). In addition, the AUC_0–24 h_ of PTX-SS-OAL NPs was 1.89 times higher than that of PTX-SS-OA NPs (Figure 4I). This was probably caused by the hydrophilic ethylene glycol contained in the PTX-SS-OA. There were certain oxidizing substances (oxygen, etc.) present in the blood circulation; therefore, the oxidative sensitivity of the prodrug nanoassemblies was a key factor influencing their fate *in vivo*. PTX-SS-OA released more PTX in plasma and showed a decrease in AUC_0–24 h_ because the presence of ethylene glycol fragments enhanced its oxidative sensitivity. In contrast, PTX-SS-OAL showed weaker oxidative sensitivity and released less PTX in plasma, resulting in a higher AUC_0–24 h_. In addition, plasma stability studies validated these findings (Figure S11G–H). After incubation of PPNAs with rat plasma for 24 h, it was observed that the PTX released from PTX-SS-OA NPs was approximately three times that of the PTX released from PTX-SS-OAL NPs. This finding was consistent with the outcomes obtained from the pharmacokinetic study.

### Biodistribution

Effective tumor accumulation was one of the key factors in the potent antitumor efficacy of prodrug nanoassemblies. Therefore, the biodistribution and intratumor accumulation of PEGylated PPNAs were measured by UPLC-MS-MS. After 1 h of administration, the tumor accumulation of PTX-SS-OA NPs was found to be lower compared to PTX-SS-OAL NPs (Figure 4J and Figure S11A–E). However, in the tumor redox microenvironment, PTX-SS-OA NPs released more PTX due to the ethylene glycol fragments, enhancing their oxidative sensitivity. This result suggested that PTX-SS-OA NPs would exert a stronger antitumor efficacy than PTX-SS-OAL NPs.

### Antitumor Efficacy and Safety

**Figure 5:**
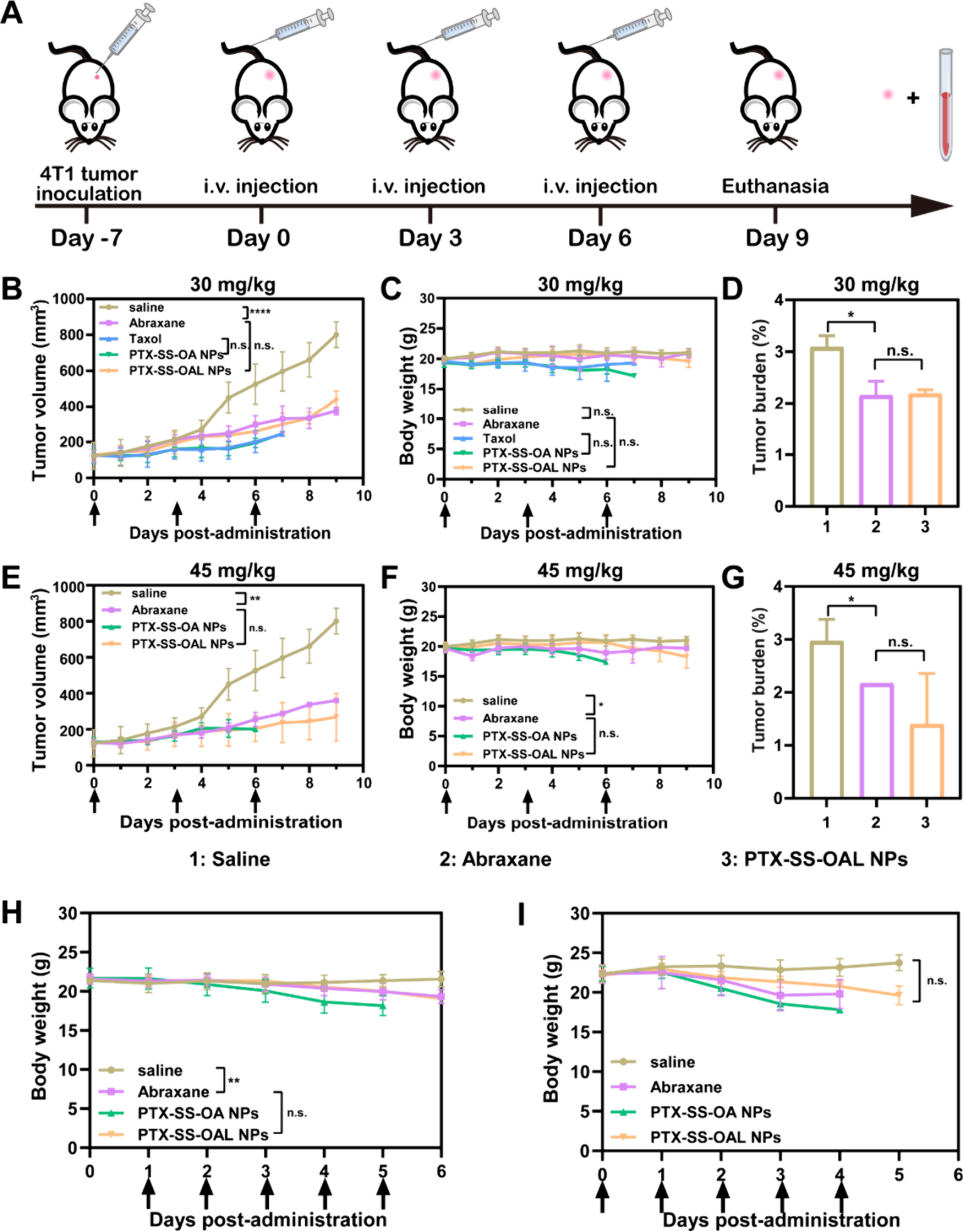
Antitumor efficacy and tolerance of PEGylated PPNAs. (A) Illustration of the administration schedule. (B–D) Tumor volume, body weight, and tumor burden at the dosage of 30 mg/kg or (E–G) 45 mg/kg. The body weight change in (H) BALB/c mice and (I) BALB/c nude mice.

To further validate the effect of ethylene glycol on the tumor growth inhibition and safety of the PPNAs, the pharmacodynamic study was conducted in a 4T1 tumor model. The administration schedule was shown in Figure 5A. Low dose (30 mg/kg, PTX equivalent concentration) and high dose (45 mg/kg, PTX equivalent concentration) were selected to assess the antitumor efficacy. Compared with the saline group, all formulations significantly inhibited the progression of the tumor. At low doses, the PTX-SS-OA NPs with excellent oxidative sensitivity exhibited remarkable tumor growth inhibition, which was comparable to Taxol. Inhibition of tumor growth by PTX-SS-OAL NPs was weaker than that by PTX-SS-OA NPs and Taxol, but it was similar to Abraxane. At high doses, PTX-SS-OA NPs and Taxol showed excellent tumor growth inhibition in all treatment groups. Although the tumor growth inhibition capacity of PTX-SS-OAL NPs was still weaker than that of PTX-SS-OA NPs and Taxol, it was slightly stronger than that of Abraxane (Figure 5B, Figure 5D–E, and Figure 5G).

In addition, the safety of the PPNAs was evaluated based on the body weight, the survival status of the mice, and blood routine. The dramatic loss of body weight and the death of the mice on the seventh day suggested that PTX-SS-OA NPs and Taxol were seriously toxic, even at a low dose (Figure 5C). In contrast, PTX-SS-OAL NPs and Abraxane caused a slight weight loss (<10%) when administered at high doses (Figure 5F). The blood routine results are displayed in Figure S12 and Figure S13. At low doses, no significant difference was shown in the main blood cell count of PTX-SS-OAL NPs and Abraxane compared to the saline group (Figure S12). When the dosage increased to 45 mg/kg, the mice intravenously administrated with Abraxane showed a decrease in white blood cells, monocytes, and neutrophils. This indicated that Abraxane caused a certain degree of myelosuppression, while PTX-SS-OAL NPs exhibited improved *in vivo* safety over Abraxane (Figure S13). The above results indicated that PTX-SS-OA NPs containing ethylene glycol showed enhanced drug release and superior antitumor efficacy, and PTX-SS-OAL NPs without ethylene glycol displayed reduced oxidative sensitivity but exhibited improved *in vivo* safety.

### Tolerance

The healthy female BALB/c mice and BALB/c nude mice were selected to investigate the tolerance of Abraxane and PPNAs. The formulations were injected at 75 mg/kg, which was higher than the dose given in the antitumor assay. Due to the excessive toxicity of Taxol, which resulted in the death of all mice at a dose of 30 mg/kg, the tolerance of Taxol was not further investigated in this experiment. The continuously body weight decrease and the death of the mice proved that PTX-SS-OA NPs exhibited poor tolerance (Figure 5H–I). The tolerance of PTX-SS-OAL NPs was comparable to Abraxane in BALB/c mice but was superior to Abraxane in BALB/c nude mice (Tables S8–S9). The reason for this difference probably was due to the resistance of HSA in BALB/c mice, which prevented Abraxane from exerting its effects. Consistent with the findings of the pharmacodynamic study, the presence of hydrophilic ethylene glycol fragments reduced the safety and tolerance of the prodrugs.

## Conclusions

Aliphatic side chains are commonly used as modification modules in self-assembled prodrugs. However, the short carbon chain lengths reduced the stability of the prodrug nanoassemblies and resulted in unfavorable pharmacokinetics, while long carbon chain lengths compromised the activation of the prodrugs. To solve the above problem, hydrophilic ethylene glycol fragments were inserted between the modification modules and the response modules, and the important effects of hydrophilic fragments on the assembly, release, and therapeutic index of the prodrugs were investigated: (i) PTX-SS-OA, containing ethylene glycol, exhibited stronger intermolecular hydrogen bonding forces, driving the stable assembly. (ii) The introduction of ethylene glycol accelerated the release of PTX from PTX-SS-OA NPs, resulting in increased redox sensitivity, boosted cytotoxicity, and enhanced antitumor efficacy. (iii) PTX-SS-OAL NPs, lacking ethylene glycol, showed limited redox sensitivity and cytotoxicity, but displayed better safety. In brief, the hydrophilic fragment is a critical determinant in modulating the therapeutic index of prodrug nanoassemblies, which is instrumental in guiding the rational design of prodrugs. Exploring the rational application of hydrophilic fragments, such as ethylene glycol, in drug delivery systems to strike a balance between efficacy and safety was of great significance for the development of highly efficient prodrug nanodelivery systems.

## Materials and Methods

### Materials

Dithiodiglycolic acid was provided by TCI Development Co., Ltd. (Shanghai, China). Oleic acid (OA), oleyl alcohol (OAL), coumarin-6 (C-6), and hydrogen peroxide (H_2_O_2_) were procured from Shanghai Aladdin Biochemical Technology Co., Ltd. (Shanghai, China). Paclitaxel (PTX), trypsin, methyl thiazolyl tetrazolium (MTT), culture medium, and glutathione (GSH) were obtained from Meilun Biotechnology Co., Ltd. (Dalian, China). DSPE-PEG_2K_ was obtained from AVT (Shanghai) Pharmaceutical Tech Co., Ltd. (Shanghai, China). 1-Ethyl-3-(3-dimethyllaminopropyl) carbodiimide hydrochloride (EDCI), 4-dimethylaminopyridine (DMAP), and 1-hydroxybenzotriazole anhydrous (HOBT) were provided by Macklin Biochemical Technology Co., Ltd. (Shanghai China). Commercial Abraxane was procured from Jiangsu Hengrui Pharmaceuticals Co., Ltd. (Jiangsu, China). Tubulin-Tracker Red was provided by Beyotime Biotechnology (Shanghai, China). Annexin V-FITC/PI apoptosis detection kit was obtained from Beijing Solarbio Science & Technology Co., Ltd. (Beijing, China). All plates and dishes were obtained from NEST Biotechnology Co., Ltd. (Wuxi, China). All other reagents used were of HPLC or analytical grade.

### Synthesis of PTX-SS-OA

A solution of 14.22 mmol of OA dissolved in toluene was introduced to 24.42 mL of ethylene glycol (0.42 mol). 1.41 mmol of p-toluenesulfonic acid was added into the above system as a catalyst. The blend was reacted for 2 h at a temperature of 110 °C. The layer containing methylbenzene was separated from the mixture, and the ethylene glycol layer was subjected to three extractions using methylbenzene. The extraction liquid was mixed with a methylbenzene layer. Finally, intermediate OA–OH was isolated through silica gel column chromatography. The yield of the intermediate was about 50%.

2,2′-Dithiodiglycolic acid (8 mmol) reacted with acetic anhydride (10 mL) under the protection of nitrogen (N_2_). After reaction for 2 h at 25 °C, the product was dissolved in 30 mL of dichloromethane (DCM). A solution comprising 0.7 mmol of DMAP and 4 mmol of OA–OH was added dropwise to the reaction mixture. It was reacted in N_2_ at 25 °C for 12 h. The unrefined intermediate was isolated by silica gel column chromatography. Following the purification, the organic solvent was evaporated, and OA-SS-COOH was obtained with a 45% yield.

A 0.6 mmol portion of OA-SS-COOH, 0.6 mmol of HOBT, 1.2 mmol of EDCI, and 0.24 mmol of DMAP were dissolved in DCM. After a 2 h activation at 0 °C, 0.25 mmol of PTX was dripped into the reaction at 25 °C and reacted for 36 h. Resulting prodrugs were then purified by preparative liquid chromatography. The yield of the purification process was 40%. Acetonitrile was employed as the mobile phase. Structural analysis and determination of molecular weight of the prodrugs were performed using ^1^H NMR spectroscopy at a frequency of 400 MHz and HRMS. High-performance liquid chromatographic determination (HPLC) was conducted to determine the purity of prodrugs.

### Synthesis of PTX-SS-OAL

After the reaction of 2,2′-dithiodiglycolic acid (8 mmol) with acetic anhydride (10 mL), the remaining acetic anhydride was dismissed through rotary evaporation. In order to dissolve the product, 30 mL of DCM was used. As a catalyst, 0.7 mmol of DMAP was dropped into the reaction. Following the addition of OAL (4 mmol), the reaction proceeded under N_2_ at a temperature of 25 °C. After the blend reacted for 12 h, the crude intermediates were purified, and the organic solvent was evaporated, resulting in the formation of the desired products (OAL-SS-COOH) with a yield of 48%.

A 0.6 mmol amount of OAL-SS-COOH, 0.6 mmol of HOBT, 1.2 mmol of EDCI, and 0.24 mmol of DMAP were activated at 0 °C for 2 h under N_2_. After the addition of 0.25 mmol PTX, the blend was reacted for 36 h to prepare prodrugs. Resulting prodrugs were then purified using preparative liquid chromatography, and the yield of the purification process was 45%. Acetonitrile was employed as the mobile phase. Structural analysis and determination of the molecular weight of the prodrugs were performed using ^1^H NMR spectroscopy at a frequency of 400 MHz and HRMS. The purity was assessed by HPLC.

### Fabrication and Characterization of PPNAs

PPNAs were prepared by a one-step nanoprecipitation method. To prepare the non-PEGylated PPNAs, anhydrous ethanol solution containing the prodrugs was added dropwise into deionized water under stirring. After that, the ethanol was evaporated. To prepare the PEGylated PPNAs, the anhydrous ethanol solution containing DSPE-PEG_2K_ and prodrugs (1/5, w/w) was added dropwise into the deionized water during magnetic stirring. Afterward, the ethanol was evaporated. For dye-labeled PPNAs, coumarin-6 was dissolved into anhydrous ethanol, and then this solution was used to dissolve the prodrugs and DSPE-PEG_2K_. The blend was added to the deionized water drop by drop. The nanoformulation underwent vacuum evaporation at 30 °C to remove the ethanol. The hydrodynamic diameter, polydispersity index (PDI), and zeta potential of PEGylated PPNAs were determined using a Zetasizer instrument (Nano ZS, Malvern Co., UK). The morphology of the PEGylated PPNAs was observed using a transmission electron microscope (TEM, Hitachi, HT7700, Japan).

### Colloidal Stability

The PEGylated PPNAs (1 mg/mL) were added to PBS (pH 7.4) containing 10% FBS (1/20, v/v). The blend was incubated at 37 °C for 12 h. The particle size change at a specified time was measured by a Zetasizer instrument. Moreover, the stability of PPNAs stored at 4 °C for 60 days or at 25 °C (room temperature) for 30 days was assessed by analyzing the size change. To further evaluate the stability of PPNAs in 0.9% NaCl, the PEGylated PPNAs (1 mg/mL) were incubated with 0.9% NaCl (1/10, v/v) at 37 °C for 24 h, and the particle size change was used as an evaluation index.

### Self-Assembly Mechanism

Non-PEGylated PPNAs were prepared by following the described procedure to validate the self-assembly mechanism. Specifically, the non-PEGylated PPNAs were added to aqueous solutions of urea, sodium dodecyl sulfate (SDS), and sodium chloride (NaCl) individually at a ratio of 1/10 (v/v). The concentration of urea, SDS, and NaCl was 0.1 mol/L. The blend was placed in an oscillator and incubated at 37 °C. Finally, the size change and PDI of the non-PEGylated PPNAs were measured by Zetasizer (Malvern). The Yinfo Cloud Computing Platform was employed to conduct the molecular simulations. The FTIR analysis was carried out by Mice Information Technology Co., Ltd. (Zhengzhou, China).

### Redox Dual-Sensitive Drug Release

To ensure accurate determination of the released concentration of PTX, it was essential to meet the sink condition. Therefore, ethanol was incorporated as a cosolvent in the release medium to ensure that the prodrug and PTX could dissolve in PBS. 0.2 mL of PEGylated PPNAs (1 mg/mL) were added into 30 mL release media which consisted of PBS, 30% ethanol (v/v), and supplemented with different concentrations of GSH (0.01 mM and 0.1 mM) or H_2_O_2_ (10 mM and 30 mM). Then, the above solution was placed in an oscillator (37 °C, 100 r/min). The PTX released from the PPNAs was quantified at specified time points by HPLC with a 227 nm detection wavelength. Besides, the PTX release behavior of PPNAs in the H_2_O_2_-free or GSH-free release medium was also investigated. To confirm the redox release mechanism, UPLC-MS-MS was used to determine the redox release intermediates.

### Cell Culture

A549 cells and KB cells were cultured in DMEM medium. 4T1 cells and L02 cells were cultured in RPMI 1640 medium. All culture media were supplemented with 10% FBS, 1% streptomycin, and penicillin. All cell lines were cultured in a 37 °C cell incubators with a 5% CO_2_ atmosphere.

### Cellular Uptake

Briefly, 4T1 cells (10^5^ cells/well) were incubated for 24 h in 24-well plates with preplaced covered lids. Then, the cells were treated with coumarin-6 solution or coumarin-6 labeled PPNAs and further incubated for 0.5 or 2 h at 37 °C. The concentration of coumarin-6 used in the formulations was 250 ng/mL. To terminate the cellular uptake, cold PBS was used to wash the cells. Then, the cells were fixed with 4% paraformaldehyde. The nuclei were counterstained with Hoechst 33342, and following the cells were washed with cold PBS. Finally, the covered lids were observed under a confocal laser scanning microscope (CLSM, C2SI, Nikon, Japan).

### Cytotoxicity Assay

The cytotoxicity of PEGylated PPNAs against murine breast tumor cells (4T1 cells), human oral epidermal tumor cells (KB cells), human nonsmall cell lung cancer cells (A549 cells), and normal human hepatic cells (L02 cells) was assessed by MTT assays. Briefly, all cell lines (2 × 10^3^ cells/well) were seeded in 96-well plates. After a 24 h incubation, the fresh medium containing different concentrations of Taxol, Abraxane, or PEGylated PPNAs was added to replace the blank medium. Meanwhile, drug-free medium was added to blank cells as a control. The MTT solution (200 μL, 0.5 mg/mL) was added to each well following a 48 h incubation. After that, the cells were further incubated for 4 h, and the medium was aspirated. Finally, 200 μL of dimethyl sulfoxide was added to dissolve the formazan. The absorbance at 490 or 570 nm was detected by a microplate reader (SYNERGY, BioTek Instruments, Inc., USA). The IC_50_ values were calculated on GraphPad Prism 8 using molar concentration and cell viability ratio as parameters. The value of the tumor-selective index (SI) was obtained by the following formula: SI = IC_50, L02_/IC_50, tumor cells_.

### Intracellular Drug Release

The cytotoxicity and antitumor efficacy of prodrug nanoassemblies were closely related to the content of the released PTX. Therefore, the amount of PTX in tumor cells was investigated. 4T1 cells (10^5^ cells/well) were seeded in 24-well plates. After a 24 h incubation, fresh RPMI 1640 medium was used to dilute the concentration of Taxol, Abraxane, and PEGylated PPNAs to 100, 200, or 500 ng/mL (PTX equivalent concentrations). Subsequently, the medicated medium was added to the plates. After a 48 h incubation, the culture medium and cells were collected for ultrasonic disruption and centrifugation to isolate the supernatant. Finally, UPLC-MS-MS was used to quantitatively analyze the content of PTX.

### Cell Apoptosis

The 4T1 cells (10^5^ cells/well) were seeded in 12-well plates. Taxol, Abraxane, or PEGylated PPNAs were diluted to 1000 nmol/L (PTX equivalent concentrations). Then, the medicated medium was added to the plates after a 24-h incubation. Following another 24 h incubation, the culture medium was collected, and the trypsin without EDTA was used to digest the cells. Before being measured by a flow cytometer (Becton Dickinson, USA), the cells were treated according to the protocol of the Annexin V-FITC/PI apoptosis detection kit. Finally, FlowJo software was used to analyze the apoptosis data.

### Microtubule Depolymerization Inhibition

The 4T1 cells (5 × 10^4^ cells/well) were seeded on covered lids in 24-well plates. The medium containing Taxol, Abraxane, or PEGylated PPNAs (PTX equivalent concentrations = 100 nmol/L) was used to replace the previous medium after incubation for 24 h. Following a further cultivation for 48 h, the medium was discarded, and the adherent cells were washed twice with cold PBS. The nuclei were stained by DAPI. The cells were fixed with immunofluorescence staining fixative for 20 min. Then, the cells were treated following the procedure of Tubulin-Tracker Red. The inhibition of microtubule depolymerization was visualized by CLSM.

### Animal Studies

The animal assays conducted in this study strictly followed the Guidelines for the Management and Use of Laboratory Animals. The study received ethical approval from the Institutional Animal Ethical Care Committee (IAEC) of Shenyang Pharmaceutical University. No unexpected or unusually high safety hazards were encountered.

### Hemolysis Assay

Blood was collected from untreated rats via the orbital route and centrifugated at 1200 rpm for 10 min. The blood cells in the bottom layer were washed and diluted with saline. Subsequently, the blood cell suspension was mixed with saline, Triton X-100 and prodrug nanoassemblies, and placed in an oscillator (37 °C, 100 r/min) for 2 h. Samples treated with saline served as the negative control, while those treated with Triton X-100 acted as the positive control. After 10 min of centrifugation at 3000 r/min, the supernatant was collected to measure the absorbance using a microplate reader, and the hemolysis percentage (HP%) was calculated by (As-An)/(Ap-An) × 100%. As, An and Ap respectively refer to the absorbance of the sample, negative control sample, and positive control sample.

### Pharmacokinetics and Plasma Stability

The pharmacokinetic characteristics of Taxol and PEGylated PPNAs were evaluated in male Sprague–Dawley rats (200–230 g). The SD rats were randomly assigned to 4 groups (*n* = 3) after fasting for 12 h. Then, Taxol and PPNAs (PTX equivalent to 5 mg/kg) were administered via the tail vein. At specified time points, blood was collected and centrifuged to obtain the plasma samples. Protein precipitation method was utilized to remove protein in plasma samples. Afterward, UPLC-MS-MS was used to analyze the concentration of free PTX and prodrugs. The pharmacokinetic characteristics were calculated with DAS 2.0 software. Moreover, the plasma stability assay was conducted by mixing the PEGylated PPNAs and the plasma (1/9, v/v). Then the blend was incubated at 37 °C. At the predetermined time point, 50 uL of plasma samples were collected, and the supernatant was obtained through protein precipitation method. The contents of PTX and prodrugs were measured by HPLC.

### Biodistribution

Female BALB/c nude mice were subcutaneously injected with a KB cell suspension (1 × 10^7^ cells/100 μL) in the right armpits. After the tumor volume reached approximately 400 mm^3^, the PEGylated PPNAs were administrated via the tail vein of the mice (PTX equivalent concentrations = 10 mg/kg). After 1 and 4 h postinjection, the mice were euthanized to dissect the tumors and the major organs (heart, liver, spleen, lung, and kidney). The content analysis of PTX and prodrugs were performed by UPLC-MS-MS.

### Antitumor Efficacy

The 4T1 cells (5 × 10^6^ cells/100 μL) were subcutaneously implanted into the right back of female BALB/c mice. Saline, Taxol, Abraxane or PEGylated PPNAs (n = 3 for each group) were intravenously administrated to the mice at doses of 30 or 45 mg/kg (PTX equivalent concentration) when the tumor volume reached approximately 120 mm^3^. All preparations were administered once every 3 days. The body weight and tumor volume of each group were recorded daily. For the tumor volume, it could be calculated using the following formula: Tumor volume (mm^3^) = (Length × Width × Width)/2. Before the fourth administration, the mice were dissected, and the isolated tumors were weighed to calculate the tumor burden. The tumor burden was calculated using the following formula: Tumor burden (%) = (M_tumor_/M_mice_) × 100 (“M_tumor_” represented the weight of the tumors, “M_mice_” represented the weight of the mice). Meanwhile, the whole blood was collected for the assessment of the blood routine.

### Tolerance

The healthy female BALB/c mice and BALB/c nude mice were used to evaluate the safety and tolerance of Abraxane and PEGylated PPNAs. Abraxane and the PPNAs were administered intravenously at a dose of 75 mg/kg (PTX equivalent concentration, n = 5 for BALB/c mice, n = 3 for BALB/c nude mice). The body weight and survival of all mice were monitored daily until the assay was terminated.

### Statistical Analysis

All the experimental data were expressed as mean value ± SD. Group comparisons were conducted by the two-tailed Student’s *t* test. n.s. (no significance) *P* > 0.05, * *P* < 0.05, ** *P* < 0.01, *** *P* < 0.001, and **** *P* < 0.0001.

## Supporting Information Available

Other experimental data: Figures: the chemical synthetic routes (Figure S1); characterizations of the prodrugs (Figure S2–S3); self-assembly mechanisms of non-PEGylated PPNAs (Figure S4); the particle sizes of non-PEGylated PPNAs (Figure S5); the changes in size distribution of non-PEGylated PPNAs in SDS (Figure S6); the redox release intermediates and their Log*P* (Figure S7); PTX release profiles in the release medium without H_2_O_2_ or GSH (Figure S8); fluorescence quantification of cellular uptake (Figure S9); the hemolysis assay of PPNAs (Figure S10); biodistribution and the stability of PEGylated PPNAs in rat plasma (Figure S11); complete blood count of BALB/C mice at doses of 30 mg/kg and 45 mg/kg (Figure S12–S13); the stability of PEGylated PPNAs in 0.9% NaCl (Figure S14). Tables: characterization of PEGylated PPNAs (Table S1); the particle sizes of non-PEGylated PPNAs (Table S2); the PDI and Zeta potential of non-PEGylated PPNAs (Table S3); IC_50_ values (Table S4); the selectivity index (Table S5); the hemolysis percentage (HP%) of the PPNAs (Table S6); pharmacokinetic parameters (Table S7); the mortality rates of the BALB/c mice and BALB/c nude mice (Table S8–S9); the HPLC conditions for determination of redox dual-sensitive drug release (Table S10); the scanning conditions of UPLC-MS-MS (Table S11); the chromatographic conditions of UPLC-MS-MS (Table S12); the HPLC conditions for determination of plasma stability (Table S13) (PDF)
