# Supplementary material for: Hydrophilic Ethylene Glycol Fragments: A Determinant Affecting the Therapeutic Index of Paclitaxel Prodrug Nanoassemblies
Source: ACS Cent Sci. 2024 Nov 20;10(12):2253–65. doi: 10.1021/acscentsci.4c01004 (PMC11672549; doi:10.1021/acscentsci.4c01004)
Supplement: Supplementary file 1 — oc4c01004_si_001.pdf [file oc4c01004_si_001.pdf]

Supplementary Information

Hydrophilic ethylene glycol fragment: A determinant affecting the therapeutic index of paclitaxel prodrug nanoassemblies

Yaqi Li[a,‡], Yixin Sun[a,‡], Qing Wang[a], Shuo Wang[a], Cuiyun Liu[a], Yuetong Huang[a], Wenxin Zhong[a], Xiyan Wang[a], Wenjing Wang[a], Shiyi Zuo[a,e], Xianbao Shi[d], Xiaohui Pu[c], Jin Sun[a,b], Zhonggui He[a,b,c], Bingjun Sun[a,b]**

[a] Department of Pharmaceutics, Wuya College of Innovation, Shenyang Pharmaceutical University, Shenyang, 110016, China.

[b] Joint International Research Laboratory of Intelligent Drug Delivery Systems, Ministry of Education, China.

[c] State Key Laboratory of Antiviral Drugs, School of Pharmacy, Henan University, N. Jinming Ave., Kaifeng 475004, China.

[d] Department of Pharmacy, The First Affiliated Hospital of Jinzhou Medical University, Jinzhou 121001, China.

[e] School of Chemical Engineering, The University of Adelaide, Adelaide, SA, 5005, Australia.

[†]These authors contributed equally to this work.

*** Corresponding Author: Prof. Zhonggui He* and Prof. Bingjun Sun*.**

Zhonggui He* − Department of Pharmaceutics, Wuya College of Innovation, Shenyang Pharmaceutical University, Shenyang, 110016, China. E-mail: hezhgui_student@aliyun.com

Bingjun Sun* − Department of Pharmaceutics, Wuya College of Innovation, Shenyang Pharmaceutical University, Shenyang, 110016, China. Email: sunbingjun_spy@sina.com

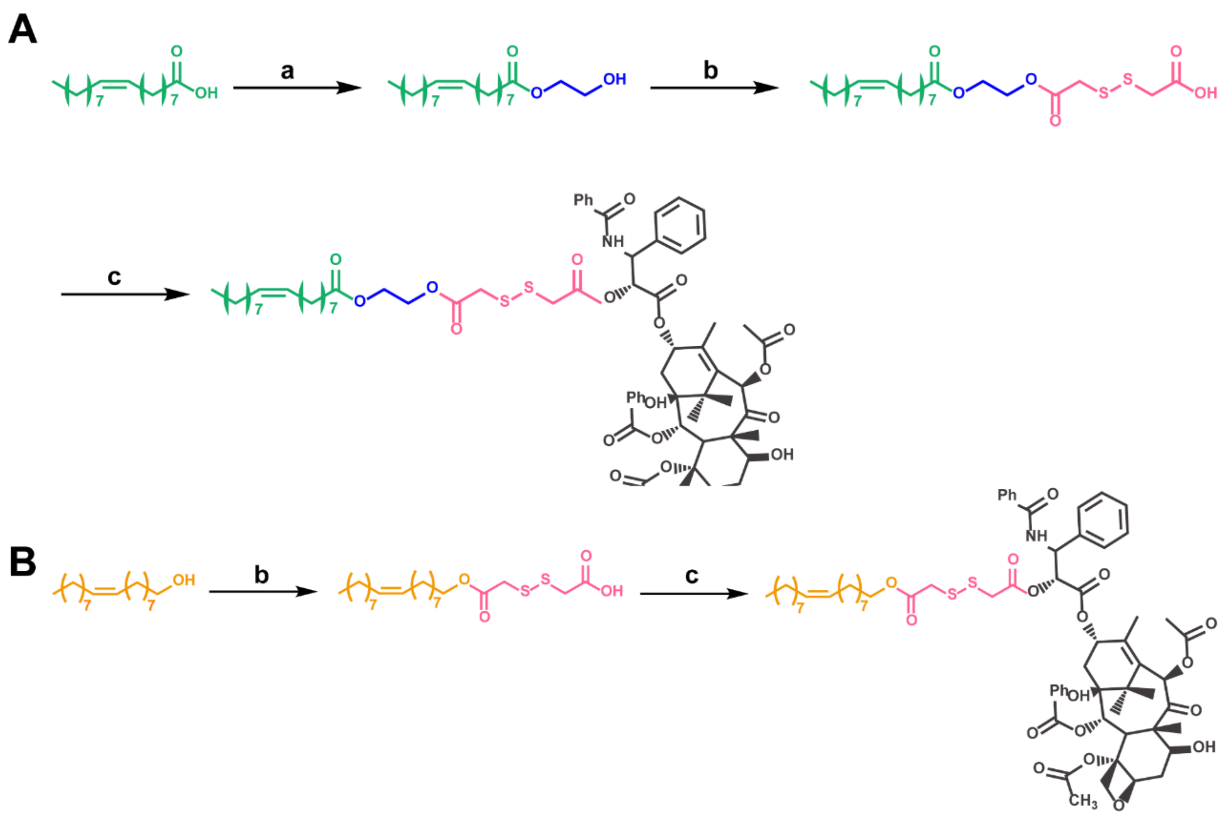

Figure S1. The chemical synthetic routes of (A) PTX-SS-OA and (B) PTX-SS-OAL. (a) Ethylene glycol, P-toluenesulfonic acid, 110°C, 2 h; (b) 2,2'-di-thiodiglycolic acid, acetic anhydride, 25°C, 2 h; DMAP, 25°C, 12 h; (c) EDCI, HOBT, DMAP, 0°C, 2 h; PTX, 25°C, 36 h.

A

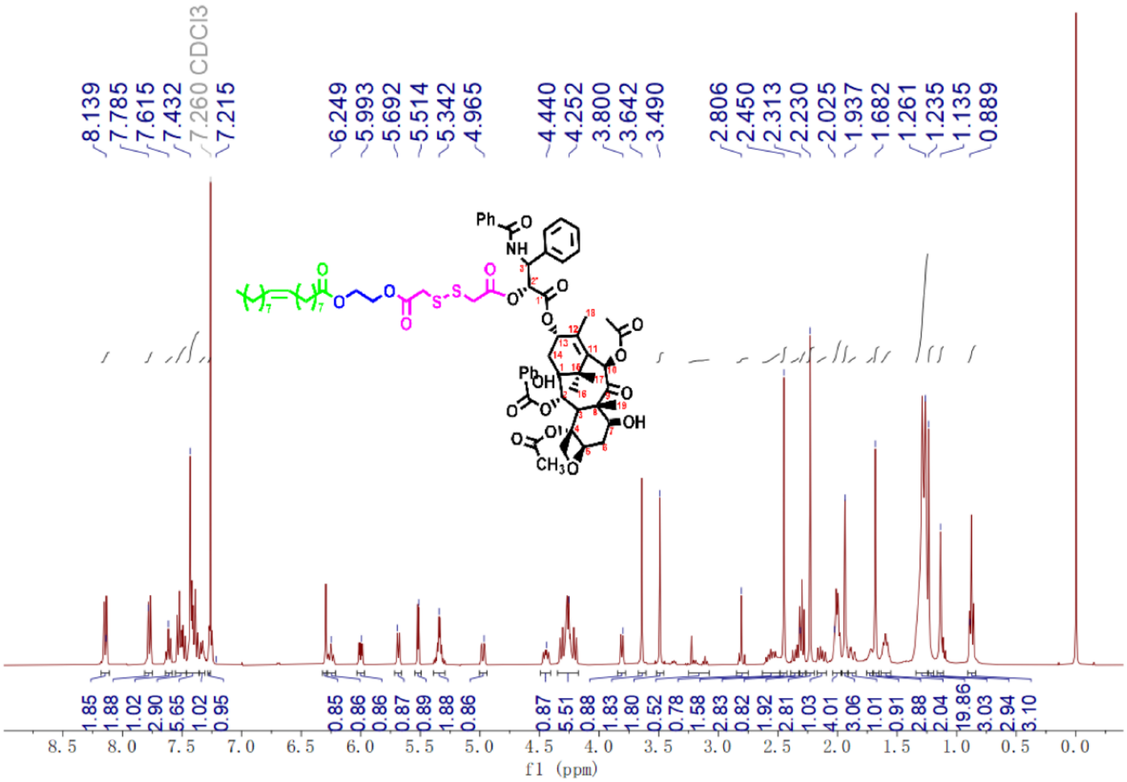

B

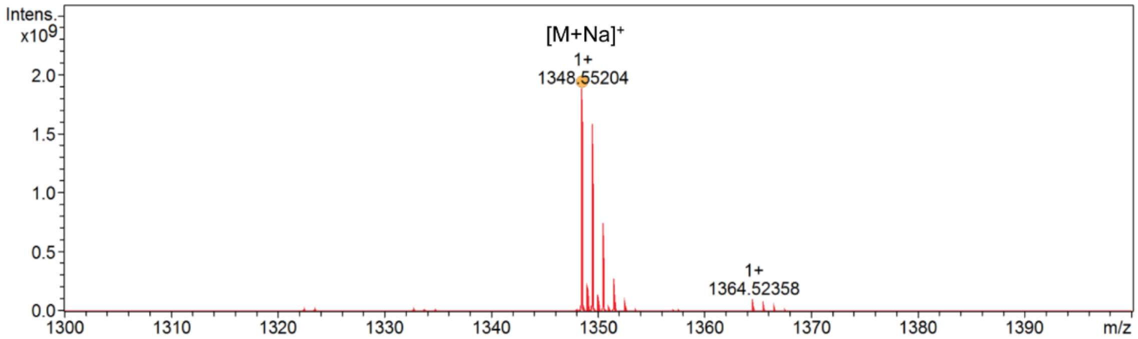

Meas. m/z	#	Ion Formula	Score	m/z	err [ppm]
1348.552039	1	C71H91NNaO19S2	100.00	1348.551892	-0.1

C

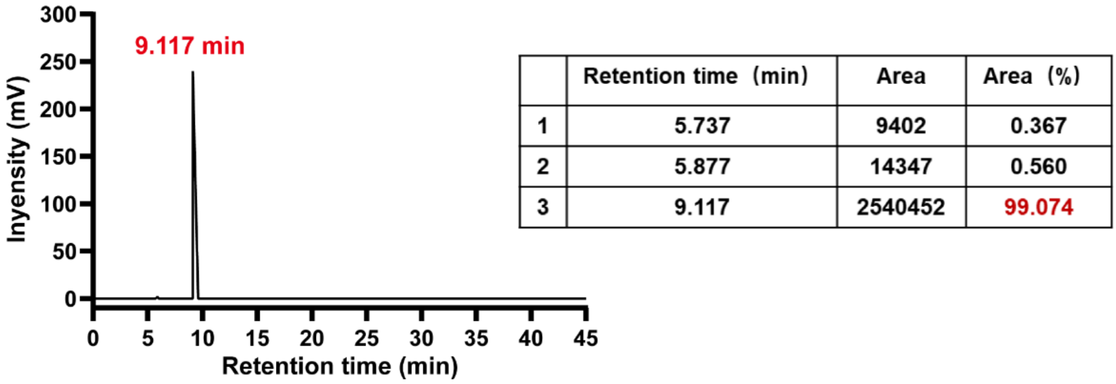

	Retention time（min）	Area	Area（%）
1	5.737	9402	0.367
2	5.877	14347	0.560
3	9.117	2540452	99.074

Figure S2. Characterizations of PTX-SS-OA. (A) ^{1}H NMR spectrum. (B) Mass spectrum. (C) The

purity.

^1H NMR (400 MHz, $CDCl_3$) δ 8.16 (d, 2H, Ar-H, *J* = 7.8 Hz), 7.76 (d, 2H, Ar-H, *J* = 7.6 Hz), 7.61 (t, 1H, Ar-H, *J* = 7.5 Hz), 7.54-7.25 (m, 10H, Ar-H), 6.23 (m, 1H, 2'-CH), 6.02 (m, 1H, -CH=CH), 5.69 (d, 1H, 3'-CH, *J* = 7.2 Hz), 5.52 (d, 1H, *J* = 2.1 Hz, -CH=CH), 5.34 (m, 2H, 10,13-CH), 4.98 (d, 1H, *J* = 8.1 Hz, 1-OH), 4.45 (m, 1H, 2-CH), 4.27 (m, 6H, -O-CH_2-CH_2-O,-CH_2-), 3.81 (d, 2H, *J* = 4.3 Hz, 5-CH), 3.64 (s, 2H, -CH_2-S-S), 3.64 (s, 2H, -S-S-CH_2), 2.45 (s, 3H, $COCH_3$), 2.22 (s, 3H, $COCH_3$), 2.37-1.85 (m, 9H, $(CH_2)_4CH$), 1.68 (s, 3H, 18-CH_3), 1.59 (m, 2H, 6-CH_2), 1.28 (m, 18H, -$(CH_2)_9$-), 1.23 (s, 3H, 19-CH_3),1.13 (s, 3H, -CH_3), 0.88 (m, 3H, -CH_3).

HRMS calcd. for $C_{71}H_{91}NO_{19}S_2$, (ESI) m/z $[M+Na]^+$ = 1348.551892.

A

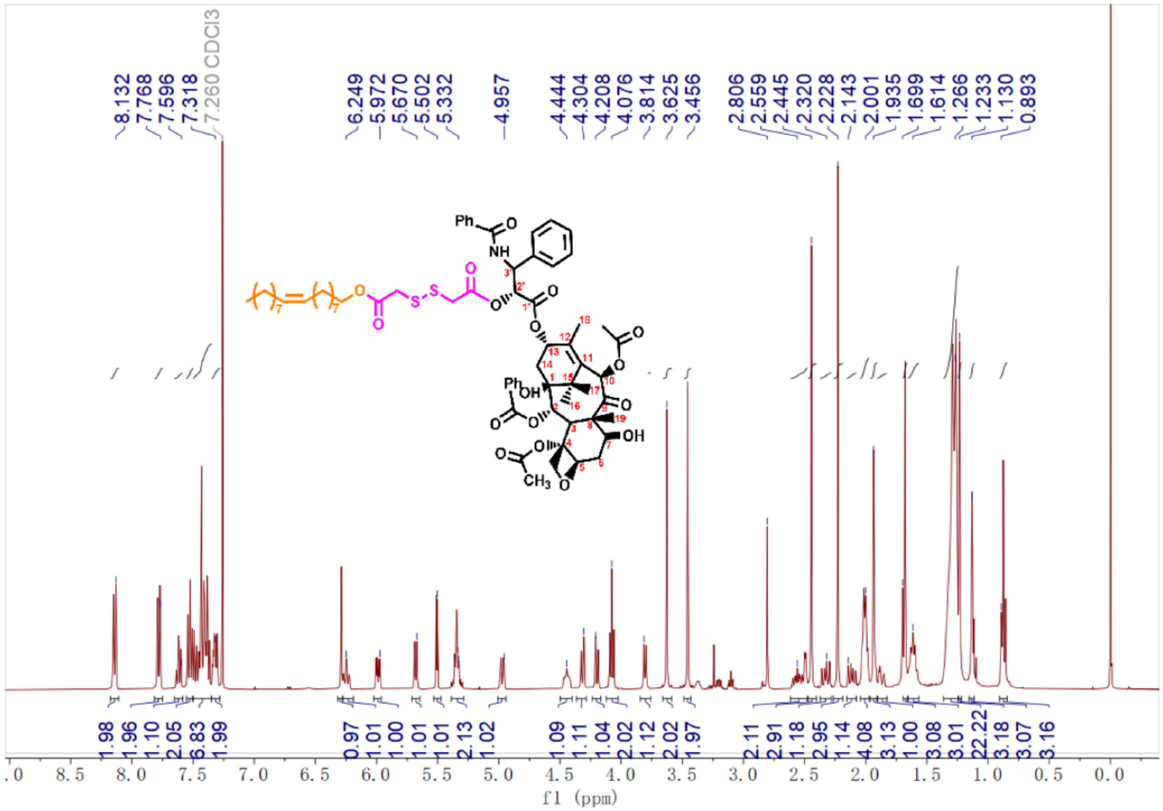

B

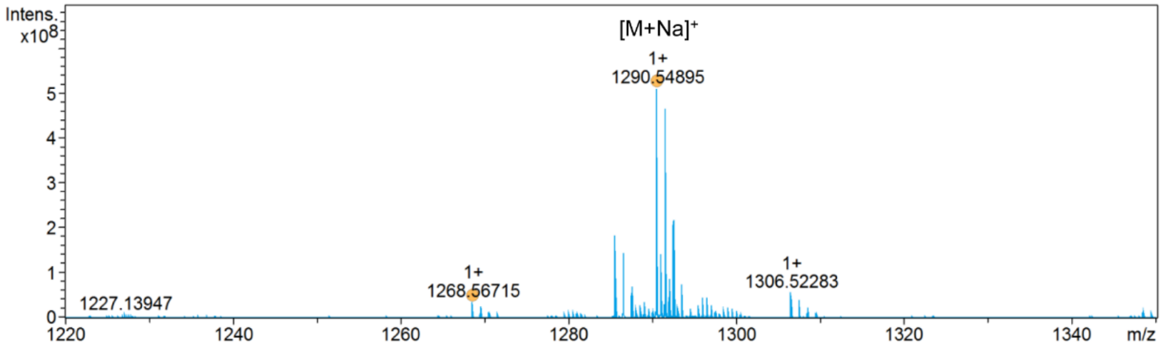

Meas. m/z	#	Ion Formula	Score	m/z	err [ppm]
1268.567148	1	C69H90NO17S2	100.00	1268.564469	-2.1
1290.548953	1	C69H89NNaO17S2	100.00	1290.546413	-2.0

C

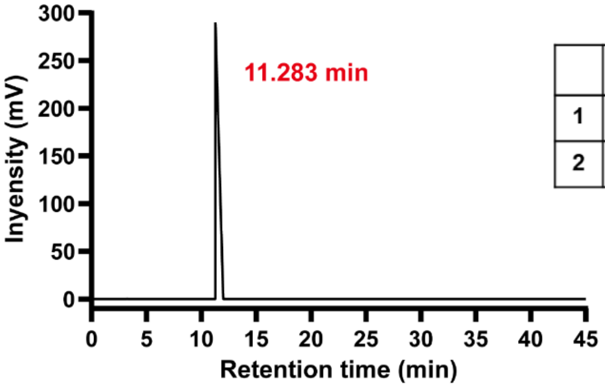

	Retention time（min）	Area	Area（%）
1	3.213	854	0.023
2	11.283	3749441	99.977

Figure S3. Characterizations of PTX-SS-OAL. (A) ^1H NMR spectrum. (B) Mass spectrum. (C) The purity.

^1H NMR (400 MHz, $CDCl_3$) δ 8.15 (d, 2H, Ar-H, *J* = 7.8 Hz), 7.77 (d, 2H, Ar-H, *J* = 7.6 Hz), 7.62 (t, 1H, Ar-H, *J* = 7.5 Hz), 7.54-7.30 (m, 10H, Ar-H), 6.24 (m, 1H, 2'-CH), 6.00 (m, 1H, -CH=CH), 5.68 (d, 1H, 3'-CH, *J* = 7.2 Hz), 5.51 (d, 1H, *J* = 2.1 Hz, -CH=CH), 5.34 (m, 2H, 10,13-CH), 4.96 (d, 1H, *J* = 8.1 Hz, 1-OH), 4.46 (m, 1H, 2-CH), 4.32-4.21 (dd, 2H, -CH_2-, *J* = 16.9, 5.2 Hz), 4.46 (t, 2H, -CH_2-), 3.81 (d, 2H, *J* = 4.3 Hz, 5-CH), 3.62 (s, 2H, -CH_2-S-S), 3.45 (s, 2H, -S-S-CH_2), 2.45 (s, 3H, COCH_3), 2.22 (s, 3H, COCH_3), 1.93 (m, 3H, -CH_2-,-CH-), 2.54-1.85 (m, 6H, $(CH_2)_3$), 1.68 (s, 3H, 18-CH_3), 1.59 (m, 2H, 6-CH_2), 1.28 (m, 18H, -$(CH_2)_9$-), 1.23 (s, 3H, 19-CH_3), 1.13 (s, 3H, -CH_3), 0.88 (m, 3H, -CH_3).

HRMS calcd. for $C_{69}H_{89}NO_{17}S_2$, (ESI) m/z $[M+Na]^+$ = 1290.546413.

Table S1. Characterization of PEGylated PPNAs.

Nanoassemblies	Size(nm)	PDI	Zeta potential (mV)	Drug loading (w/w, %)
PTX-SS-OA NPs	75.12 ± 2.10	0.18 ± 0.01	-20.80 ± 0.15	51.54
PTX-SS-OAL NPs	72.92 ± 1.27	0.19 ± 0.04	-20.20 ± 0.46	53.89

PPNAs, PTX prodrug nanoassemblies; PDI, polydispersity index.

Drug loading = ($m_{prodrug} \times M_{PTX} / M_{prodrug}$)/($m_{prodrug} + m_b$). ("$m_{prodrug}$" represents the weight of the prodrugs, "m_b" represents the weight of the DSPE-PEG_{2K}, "M_{PTX}" represents the molecular weight of PTX, "$M_{prodrug}$" represents the molecular weight of prodrugs).

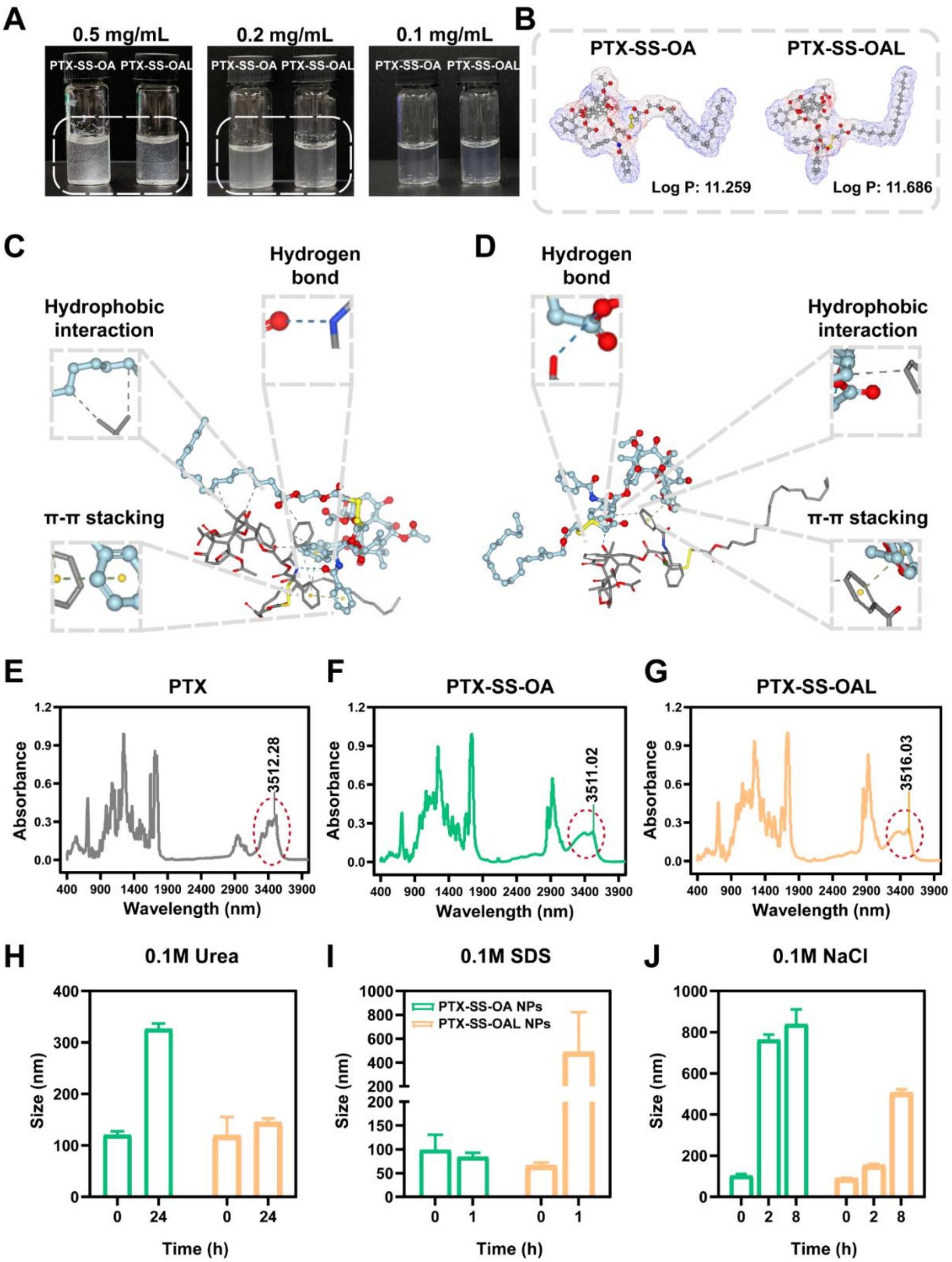

Figure S4. Self-assembly mechanisms of non-PEGylated PPNAs. (A) The appearance of PPNAs. (B) Log*P* values of two prodrugs. Molecular conformations of (C) PTX-SS-OA and (D) PTX-SS-OAL during the self-assembly process. (E-G) The FTIR spectroscopy results. The variation of the particle size of non-PEGylated PPNAs after co-incubated with (H) urea, (I) SDS or (J) NaCl.

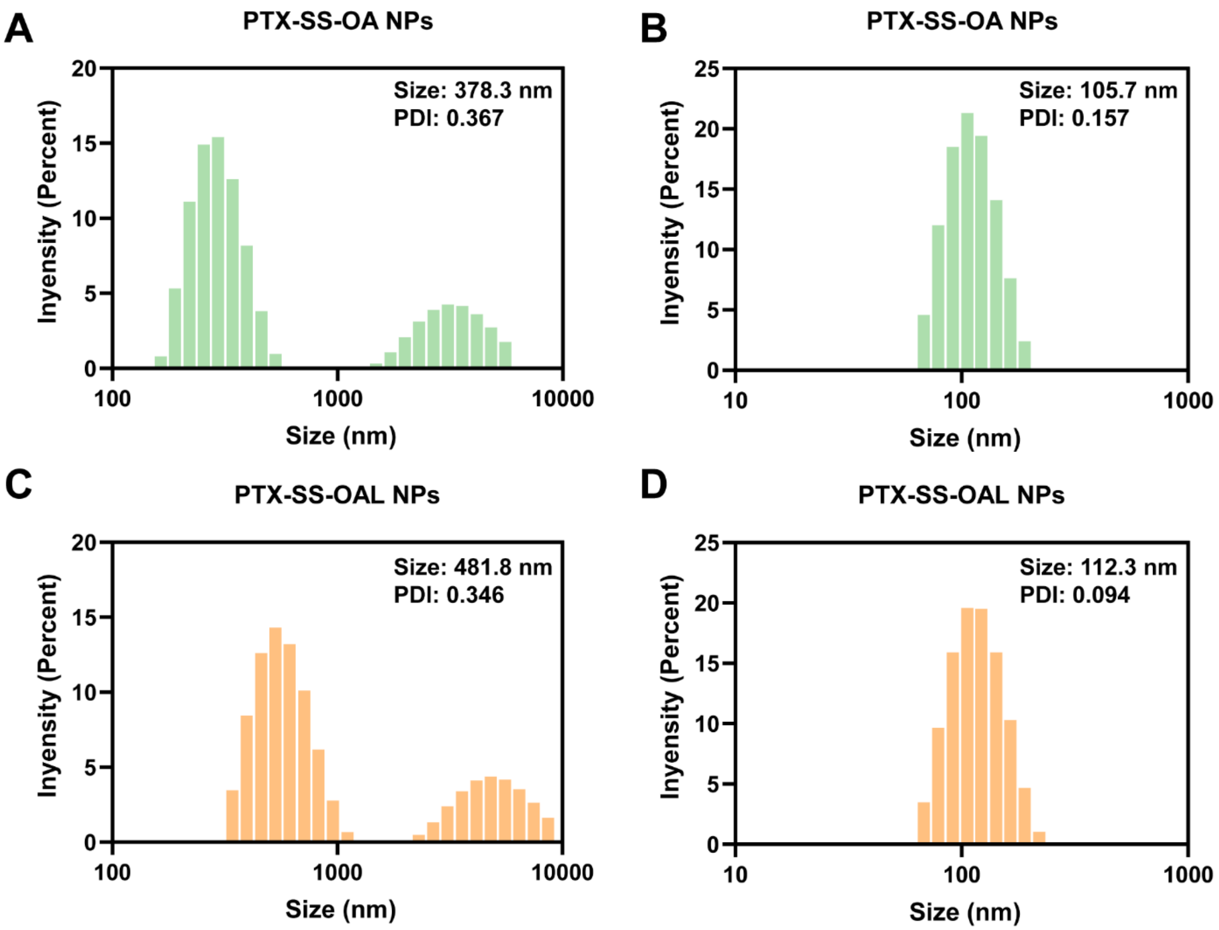

Figure S5. The particle sizes of non-PEGylated PTX-SS-OA NPs at concentrations of (A) 0.2 mg/mL and (B) 0.1 mg/mL. The particle sizes of non-PEGylated PTX-SS-OAL NPs at concentrations of (C) 0.2 mg/mL and (D) 0.1 mg/mL.

Table S2. The particle sizes of non-PEGylated PTX-SS-OA NPs and non-PEGylated PTX-SS-OAL NPs.

Concentration	PTX-SS-OA NPs	PTX-SS-OAL NPs
0.50 mg/mL	Obvious precipitation	Obvious precipitation
0.20 mg/mL	378.30 ± 8.44	481.80 ± 26.07
0.10 mg/mL	105.70 ± 1.54	112.30 ± 0.85

Table S3. The PDI and Zeta potential of non-PEGylated PTX-SS-OA NPs and non-PEGylated PTX-SS-OAL NPs at the concentrations of 0.20 and 0.10 mg/mL.

Concentration	PDI		Zeta potential (mV)	
	PTX-SS-OA NPs	PTX-SS-OAL NPs	PTX-SS-OA NPs	PTX-SS-OAL NPs
0.10 mg/mL	0.37 ± 0.11	0.35 ± 0.06	Precipitation	Precipitation
0.20 mg/mL	0.15 ± 0.01	0.09 ± 0.04	-16.50 ± 0.78	-16.10 ± 0.20

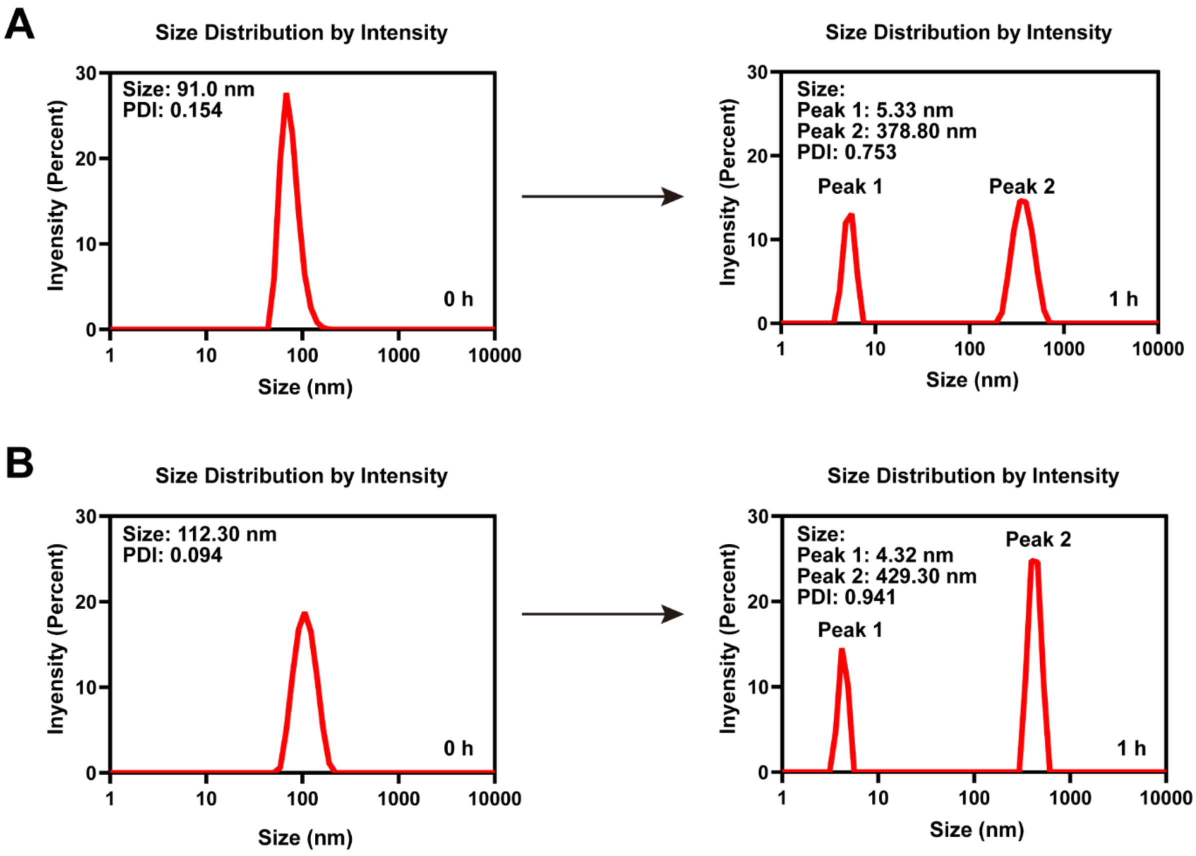

Figure S6. The changes in size distribution of (A) non-PEGylated PTX-SS-OA NPs and (B) non-PEGylated PTX-SS-OAL NPs in SDS.

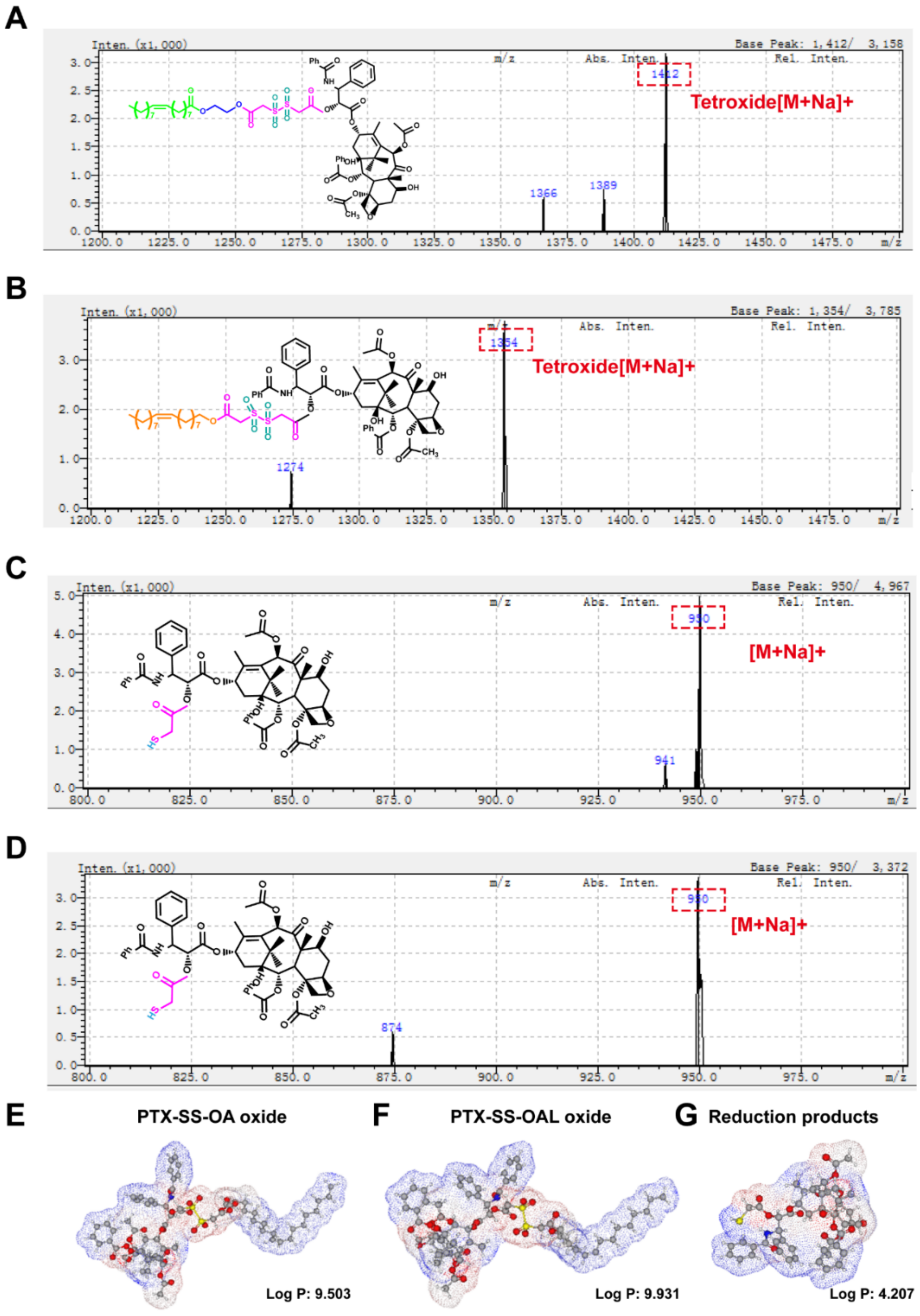

Figure S7. The oxidation intermediates of (A) PTX-SS-OA NPs and (B) PTX-SS-OAL NPs. The reduction intermediates of (C) PTX-SS-OA NPs and (D) PTX-SS-OAL NPs. The calculated Log*P* of oxidation intermediates of (E) PTX-SS-OA NPs and (F) PTX-SS-OAL NPs. (G) The calculated Log*P* of the reduction intermediates.

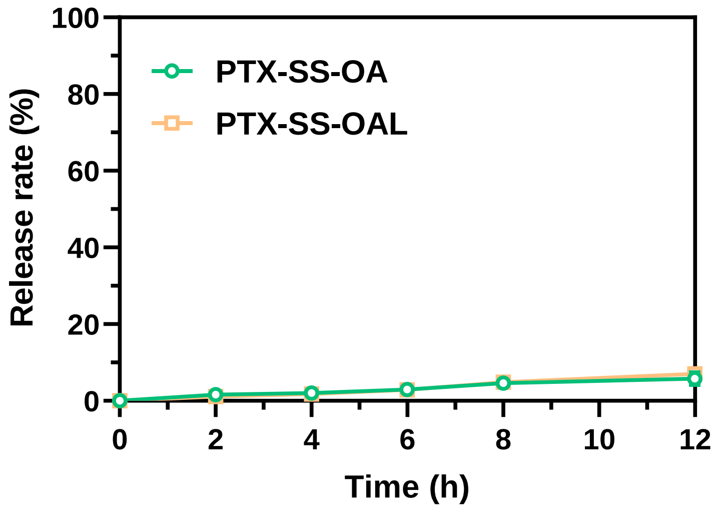

Figure S8. PTX release profiles in the release medium without H_2O_2 or GSH.

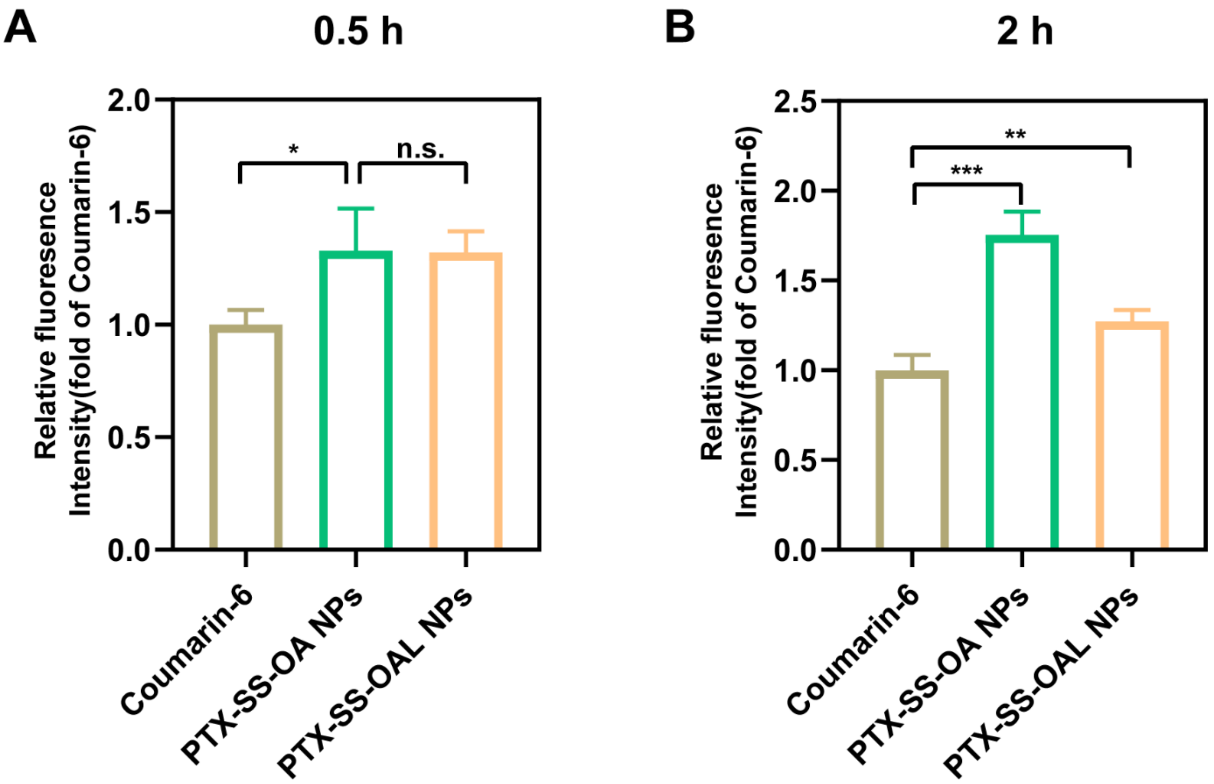

Figure S9. Fluorescence quantification of cellular uptake at (A) 0.5 and (B) 2 h. Data are presented as mean ± SD ($n = 3$). n.s. (no significance) $P > 0.05$, * $P < 0.05$, ** $P < 0.01$, *** $P < 0.001$, and **** $P < 0.0001$ by two-tailed Student's t-test.

Table S4. IC_{50} values (nmol/L) of Taxol, Abraxane and PEGylated PPNAs against three tumor cell lines and one normal cell line.

Cell lines	Taxol	Abraxane	PTX-SS-OA NPs	PTX-SS-OAL NPs
4T1	3.75 ± 0.22	18.50 ± 0.18	90.57 ± 3.06	150.20 ± 42.24
KB	1.32 ± 0.04	3.89 ± 0.24	23.78 ± 1.42	55.06 ± 0.28
A549	3.33 ± 2.49	4.04 ± 0.58	45.48 ± 4.25	133.0 ± 8.48
L02	24.35 ± 3.19	32.65 ± 0.89	870.40 ± 34.14	4804.0 ± 316.08

Table S5. The selectivity index (SI) of Taxol, Abraxane and PEGylated PPNAs between L02 cells and tumor cells.

Cell lines	Taxol	Abraxane	PTX-SS-OA NPs	PTX-SS-OAL NPs
4T1	6.49	1.76	9.61	31.98
KB	18.50	8.40	36.60	87.25
A549	7.32	8.07	19.14	36.12

SI = ($IC_{50,\ L02}$ / $IC_{50,\ tumor\ cells}$). ("$IC_{50,\ L02}$ and $IC_{50,\ tumor\ cells}$" represent the IC_{50} of L02 cells and 4T1, A549 or KB cells).

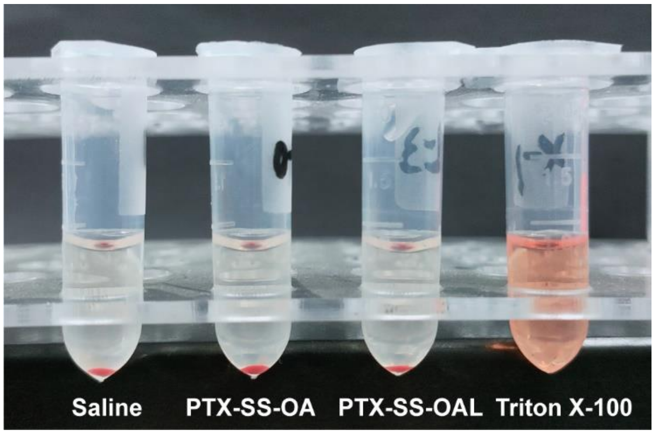

Figure S10. The hemolysis assay of PPNAs.

Table S6. The hemolysis percentage (HP%) of the PPNAs.

Preparations	Saline	PTX-SS-OA NPs	PTX-SS-OAL NPs	Triton X-100
HP (%)	Negative control	0.28% ± 0.01%	0.24% ± 0.01%	Positive control

HP (%) = (As-An) / (Ap-An) ×100%, ("As, An and Ap" respectively refer to the absorbance of the sample, negative control sample and positive control sample.

Data are presented as n = 3.

Table S7. Pharmacokinetic parameters of Taxol and PEGylated PPNAs.

Formulations	Determined	$AUC_{0\text{-}24\,h}$ (nmol/mL·h)	$MRT_{0\text{-}24\,h}$(h)	C_{max} (nmol/mL)
Taxol	PTX	11.49 ± 2.54	2.45 ± 0.50	21.45 ± 1.18
PTX-SS-OA NPs	PTX-SS-OA	82.95 ± 31.70	7.91 ± 11.42	101.36 ± 27.25
	PTX	10.55 ± 2.52	3.75 ± 0.92	5.22 ± 2.33
PTX-SS-OAL NPs	PTX-SS-OAL	168.96 ± 45.94	1.58 ± 0.25	122.11 ± 16.92
	PTX	10.09 ± 2.60	3.37 ± 0.59	5.12 ± 0.28

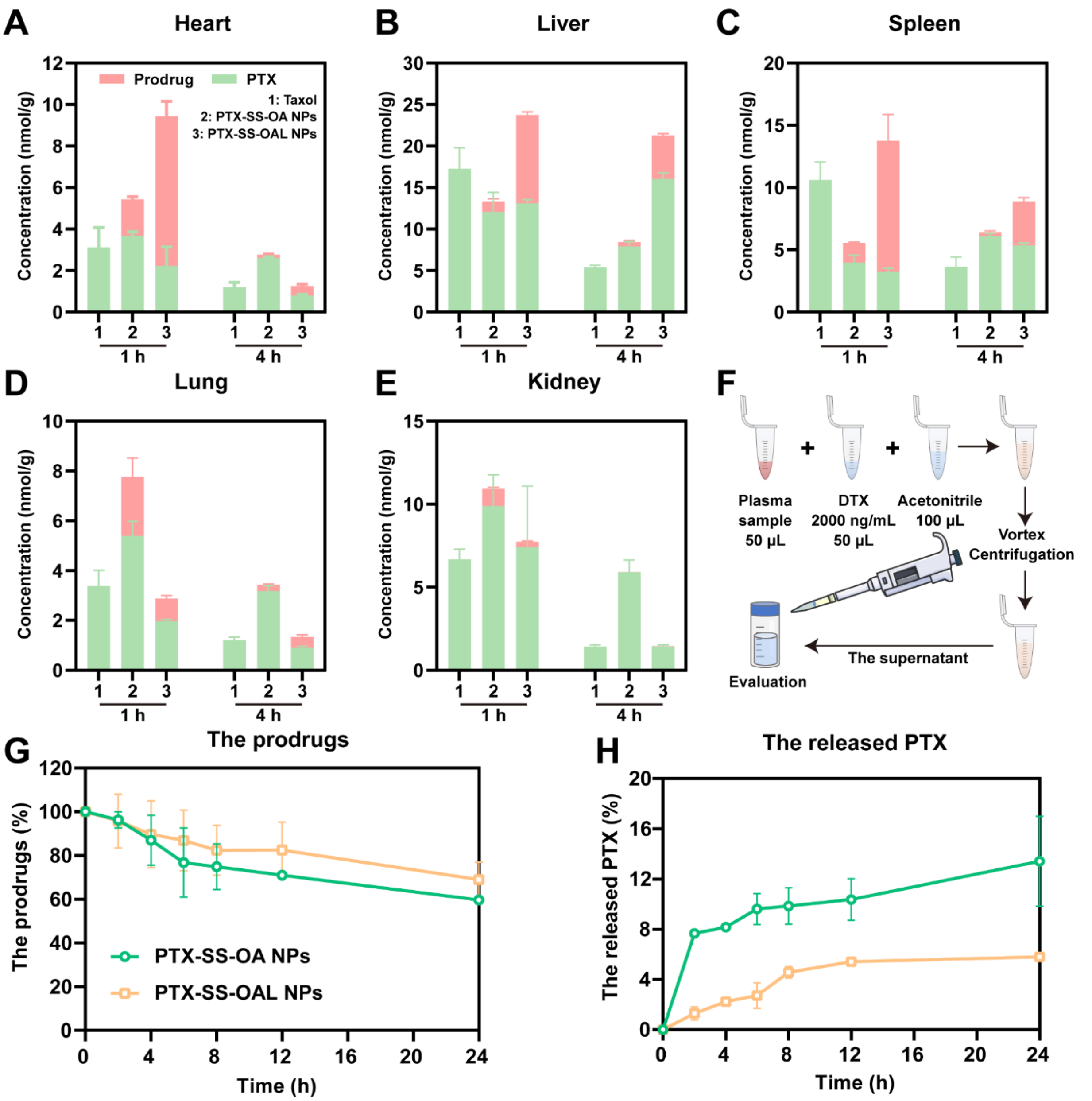

Figure S11. Biodistribution of PEGylated PPNAs in (A) heart, (B) liver, (C) spleen, (D) lung and (E) kidney. (F) The process of protein precipitation method. The stability of PPNAs in rat plasma. (G) The profiles of the prodrugs and (H) the released PTX. Data are presented as mean ± SD (n = 3).

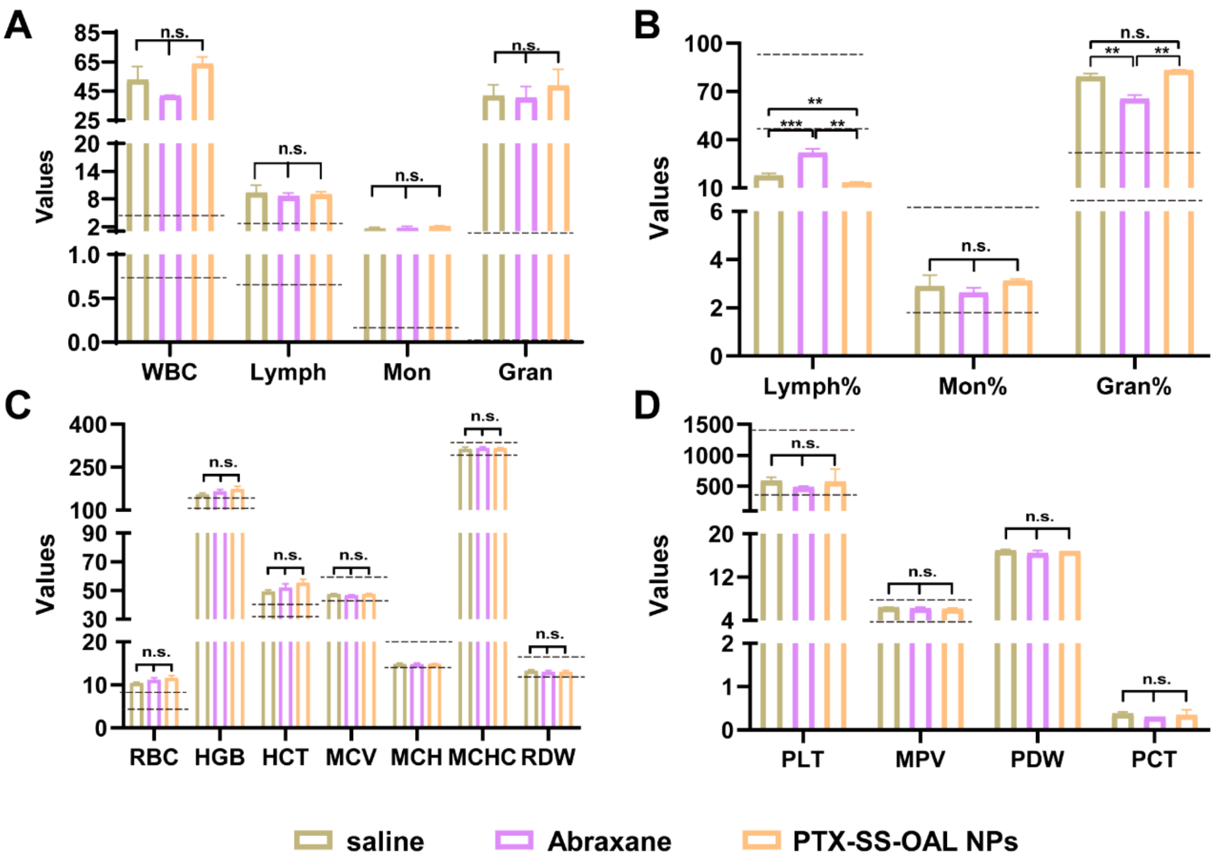

Figure S12. Safety analysis and complete blood count of BALB/C mice at doses of 30 mg/kg. (A) WBC: white blood cell count (10^9 L^{-1}), Lymph: lymphocyte count (10^9 L^{-1}), Mon: monocyte count (10^9 L^{-1}), Gran: granulocyte count (10^9 L^{-1}). (B) Lymph%: lymphocyte percentage (%), Mon%: monocyte percentage (%), Gran%: granulocyte percentage (%). (C) RBC: red blood cell count (10^{12} L^{-1}), HGB: heoglobin (g/L), HCT: hematocrit (%), MCV: mean red blood cell volume (fL), MCH: mean corpuscular hemoglobin (pg), MCHC: mean corpuscular hemoglobin concentration (g/L), RDW: red cell distribution width (%), (D) PLT: platelet count (10^9 L^{-1}), MPV: average platelet volume (fL), PDW: platelet distribution width, PCT: platelet hematocrit (%). Data are presented as mean ± SD (n = 3). n.s. (no significance) $P > 0.05$, * $P < 0.05$, ** $P < 0.01$, *** $P < 0.001$, and **** $P < 0.0001$ by two-tailed Student's t-test.

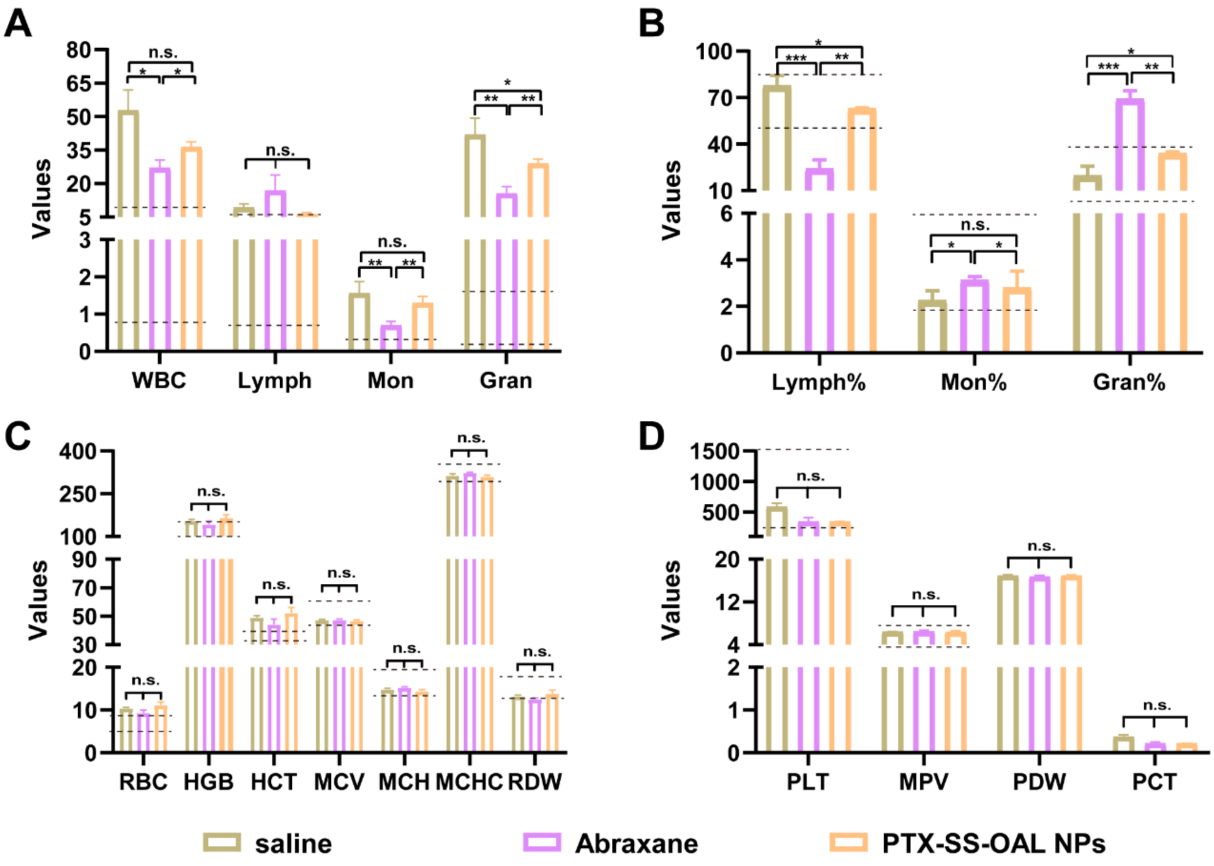

Figure S13. Safety analysis and complete blood count of BALB/C mice at doses of 45 mg/kg. (A) WBC: white blood cell count (10^9 L^{-1}), Lymph: lymphocyte count (10^9 L^{-1}), Mon: monocyte count (10^9 L^{-1}), Gran: granulocyte count (10^9 L^{-1}). (B) Lymph%: lymphocyte percentage (%), Mon%: monocyte percentage (%), Gran%: granulocyte percentage (%). (C) RBC: red blood cell count (10^{12} L^{-1}), HGB: heoglobin (g/L), HCT: hematocrit (%), MCV: mean red blood cell volume (fL), MCH: mean corpuscular hemoglobin (pg), MCHC: mean corpuscular hemoglobin concentration (g/L), RDW: red cell distribution width (%), (D) PLT: platelet count (10^9 L^{-1}), MPV: average platelet volume (fL), PDW: platelet distribution width, PCT: platelet hematocrit (%). Data are presented as mean ± SD (n = 3). n.s. (no significance) $P > 0.05$, * $P < 0.05$, ** $P < 0.01$, *** $P < 0.001$, and **** $P < 0.0001$ by two-tailed Student's t-test.

Table S8. The mortality rates of the BALB/c mice.

Formulations	Number of dead mice			Mortality rate (%)
	Day 4	Day 5	Day 6	
Saline	0	0	0	0
Abraxane	0	0	0	0
PTX-SS-OA NPs	0	1	5	100
PTX-SS-OAL NPs	0	0	0	0

Data are presented as n = 5.

Table S9. The mortality rates of the BALB/c nude mice.

Formulations	Number of dead mice			Mortality rate (%)
	Day 4	Day 5	Day 6	
Saline	0	0	0	0
Abraxane	0	1	3	100
PTX-SS-OA NPs	0	2	3	100
PTX-SS-OAL NPs	0	0	0	0

Data are presented as n = 3.

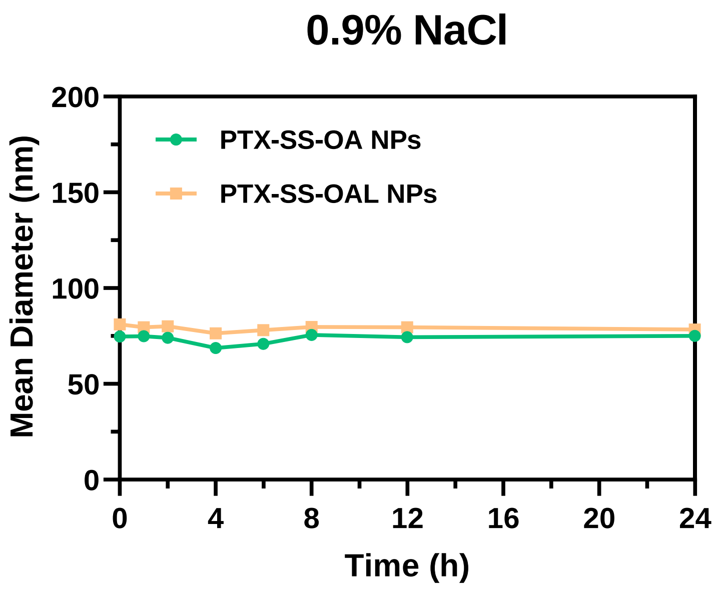

Figure S14. The stability of PEGylated PPNAs in 0.9% NaCl.

Table S10. The HPLC conditions for determination of redox dual-sensitive drug release.

Chromatographic column	Column temperature	Detection wavelength	Mobile phase Acetonitrile: water (%)	Velocity of flow (mL/min)
Welch AQ-C18	25 ± 5℃	227 nm	65 : 35	1

Table S11. The scanning conditions of UPLC-MS-MS.

Compounds	Parent (m/z)	Daughter (m/z)	Cone (V)	Collision (V)
PTX	876.6	308.2	40	35
DTX	830.5	304.1	40	30
PTX-SS-OA	1348.7	780.1	60	40
PTX-SS-OAL	1290.6	722.2	60	40

Table S12. The chromatographic conditions of UPLC-MS-MS.

**Chromatographic column**	**Organic phase （98%）**	**Water phase （2%）**	**Velocity of flow (mL/min)**	**Injection volume （μL）**
Phenomenex Kinetex® XB-C18 column	Acetonitrile	Water + 0.1% formic acid	0.2	5

Table S13. The HPLC conditions for determination of plasma stability.

**Time (min)**	**Acetonitrile (%)**	**Water (%)**
0	60	40
8.0	60	40
8.1	100	0
28.0	100	0
30.0	60	40
33.0	60	40
